# Associations of leucocyte subtypes and platelet parameters with kidney cancer risk in the UK Biobank cohort

**DOI:** 10.1186/s12950-025-00458-6

**Published:** 2025-08-11

**Authors:** Sofia Christakoudi, Konstantinos K. Tsilidis, Marc J. Gunter, Elio Riboli

**Affiliations:** 1 Department of Epidemiology and Biostatistics, School of Public Health, Imperial College London, White City Campus, 90 Wood Lane, London, W12 0BZ UK; 2 Department of Hygiene and Epidemiology, University of Ioannina School of Medicine, Ioannina, Greece

**Keywords:** Neutrophils, Lymphocytes, Platelets, MPV, PDW, Hip index, Kidney cancer, Prospective

## Abstract

**Background:**

Kidney cancer is related to obesity and inflammation and platelets are involved in thrombo-inflammation, but the prospective associations of individual leucocyte subtypes and platelet parameters with kidney cancer risk are unclear.

**Methods:**

Using data from the UK Biobank cohort and multivariable Cox proportional hazards models, we obtained hazard ratios (HR per one standard deviation increase) with 95% confidence intervals (95%CI) for the mutually adjusted associations of inflammatory markers and platelet parameters (log-transformed), and allometric obesity indices (body mass index (BMI), a body shape index (ABSI), hip index) with kidney cancer risk (overall, by sex, and by follow-up time with a cut-off at 6 years).

**Results:**

During a mean follow-up of 10.4 years, 1086 kidney cancers were ascertained in 396,482 participants. Conditional on each other and covariates, neutrophil count (HR = 1.12; 95%CI = 1.04 − 1.20), C-reactive protein (HR = 1.11; 95%CI = 1.04 − 1.19), platelet count (HR = 1.18; 95%CI = 1.10 − 1.27), platelet distribution width (HR = 1.16; 95%CI = 1.09 − 1.24), and BMI (HR = 1.22; 95%CI = 1.14 − 1.30) were positively associated, while lymphocyte count (HR = 0.90; 95%CI = 0.84 − 0.96) and hip index (HR = 0.88; 95%CI = 0.83 − 0.93) were inversely associated with kidney cancer risk in participants overall, but there was little evidence for an association with ABSI (HR = 1.05; 95%CI = 0.99 − 1.12). There were no major sex differences, but the positive association with C-reactive protein was observed only for shorter follow-up time (HR = 1.26; 95%CI = 1.14 − 1.38; p-follow-up = 0.0006).

**Conclusions:**

Our findings support two separate longer-acting pathways in kidney cancer development– a pathway related to general rather than abdominal obesity and an immune-cell-related pathway involving neutrophils assisted by activated platelets, as well as a cancer-induced thrombo-inflammation closer to kidney cancer diagnosis.

**Supplementary Information:**

The online version contains supplementary material available at 10.1186/s12950-025-00458-6.

## Background

Kidney cancer is ranked 16th according to incidence in women and men combined (2.2% of all incident cancer cases in GLOBOCAN 2020) and reaches 9th (2.7%) for men [1]. It is, however, one of the thirteen obesity-related cancers [2] and shows clear positive associations with body mass index (BMI) in women and men [3–6]. One of the mechanisms proposed to explain how obesity promotes the development of kidney cancer is through chronic inflammation triggered by hypertrophy-related adipocyte death, which is associated with secretion of pro-inflammatory cytokines conferring a pro-inflammatory phenotype to immune cells [7]. This process is further supported by cytokine production and recruitment of inflammatory cells by kidney cancer cells [8]. Correspondingly, BMI is positively associated with C-reactive protein (CRP) and inflammatory cytokine levels [9, 10]. CRP has also been positively associated with kidney cancer risk in prospective studies [11]. Although it is generally accepted that chronic inflammation is involved in the development and progression of kidney cancer [8, 12], surprisingly little is known about the prospective associations of leucocyte (white blood cell) subtypes with kidney cancer risk. A study in the UK Biobank cohort, examining women and men jointly, reported positive associations of the systemic inflammation index (calculated as (neutrophils*platelets)/ lymphocytes), the neutrophil-to-lymphocyte ratio, and the platelet-to-lymphocyte ratio, and an inverse association of the lymphocyte-to-monocyte ratio with kidney cancer risk [13]. The associations with individual leucocyte subtypes, however, are unclear.

Platelets are also involved in inflammatory processes and co-operate with neutrophils to facilitate cancer development [14, 15]. Correspondingly, a large prospective nested case-control study has shown higher kidney cancer risk in individuals with very high platelet count (above the 90th centile or with clinically defined thrombocytosis above 450*10^9^/L), with a similar risk for follow-up between 5 and 10 years and between 18 and 24 months after platelet count measurement and a stronger association for follow-up within the first six months after platelet count measurement [16]. Little is known, however, about prospective associations of platelet size reflected in mean platelet volume (MPV) and platelet distribution width (PDW) with kidney cancer risk. A few small-scale case-control studies have reported lower MPV at kidney cancer diagnosis [17], but there are no studies, to our knowledge, examining prospective associations with MCV or PDW.

Body shape evaluated with allometric indices, which factor out the positive correlations of waist and hip circumferences with body mass index (BMI) and height prior to the statistical analysis, is associated with inflammatory markers and with cancer independent of body size [9, 18]. In UK Biobank participants, waist size (reflected in the allometric “a body shape index”, ABSI) was positively associated with kidney cancer risk, as well as with leucocyte subtypes and CRP, while hip size (reflected in the allometric hip index, HI) was inversely associated with kidney cancer risk and with lymphocyte count [9, 18]. It is unclear, however, whether body shape and size would retain their associations with kidney cancer risk after accounting for inflammatory factors and platelet parameters.

In this study, using data from the UK Biobank cohort, with a longer follow-up compared to the earlier reports [11, 13, 18], we have examined the associations of inflammatory markers (represented by individual leucocyte subtype counts − neutrophil, monocyte, and lymphocyte counts, and CRP) and platelet parameters (platelet count, MPV, and PDW) with kidney cancer risk. We have re-examined associations of allometric obesity indices with kidney cancer risk with a larger number of cases and have evaluated the associations of the exposures using mutually adjusted combined models. We have compared traditional and allometric body shape indices. We have examined heterogeneity between women and men and according to follow-up time.

## Methods

### Study population

UK Biobank includes some half a million participants from England, Scotland, and Wales aged 40 to 70 years at recruitment (years 2006 to 2010) [19]. As in our previous study [18], we included participants with self-reported white ancestry and excluded participants with a prevalent cancer at recruitment, a mismatch between the genetic and self-reported sex, and pregnant women, and additionally excluded participants using antihemorrhagic agents (total excluded up to this stage 66,018 (13.1%) cohort participants) (Supplementary Table S1). We used this extended dataset for sensitivity analyses. For the main analysis dataset (complete-exposures dataset), we further excluded 39,869 (7.9%) cohort participants with missing anthropometric measurements, leucocyte counts, platelet parameters, or CRP measurements, excluding in total 105,887 (21.1%) cohort participants. In a sensitivity analysis, we further excluded 13,985 cohort participants (3.5% of the main dataset) with missing any covariate (complete-covariates dataset).

### Kidney cancer ascertainment

Information for cancer diagnosis in UK Biobank is obtained from the national cancer registries of the United Kingdom. Kidney cancer cases were defined as the first primary cancer diagnosed after recruitment with code C64 from the 10th version of the International Statistical Classification of Diseases (ICD10) and behavioural code 3 (malignant, primary site) or 5 (malignant, microinvasive) [18]. Follow-up was censored at the date of diagnosis for first primary kidney cancer with behavioural code 6 (malignant, metastatic site), or 9 (malignant, uncertain whether primary or metastatic site), or missing or with rare morphology (codes 8800, 8830, 8890, 8964), and for first primary cancer outside the kidney (except skin basocellular carcinomas). For participants remaining cancer-free, follow-up was censored at the earlier of the date of death or the last date of complete cancer registry (31 December 2016 for Wales; 31 March 2020 for England and Scotland). In a sensitivity analysis, we examined all primary incident kidney cancers as outcome (irrespective of the order of diagnosis), continuing follow-up after diagnosis for cohort participants with incident cancer in another location and defining exit time as the earliest of the date of diagnosis of a primary incident kidney cancer, or death, or last complete follow-up.

### Exposure measurements

Blood samples were obtained at recruitment and were taken throughout the day and irrespective of fasting status. Blood cell parameters were measured within 24 h of blood draw on Beckman Coulter LH750 analysers [20]. CRP was measured in serum on Beckman Coulter AU5800 analysers [21].

Anthropometric measurements were obtained by UK Biobank technicians at recruitment according to pre-defined protocols [22]. Waist circumference (WC) was measured at the natural indent or the umbilicus and hip circumference (HC) was measured at the widest point. BMI was calculated as Weight(kg) * Height(m)^−2^. Waist-to-hip ratio (WHR) was calculated as WC(cm) divided by HC(cm). ABSI was calculated as 1000 * WC(m) * Weight(kg)^−2/3^ * Height(m)^5/6^ [23]. Waist-to-hip index (WHI) was calculated as WHR * Weight(kg)^−1/4^ * Height(cm)^1/2^ [18]. HI in women was calculated as HC(cm) * Weight(kg)^−0.482^ * Height(cm)^0.310^ [24]. HI in men was calculated as HC(cm) * Weight(kg)^−2/5^ * Height(cm)^1/5^ [9]. We used coefficients based on UK Biobank data for HI in men to avoid the negative correlation with BMI for HI calculated with the original formula [18].

### Statistical analysis

We used STATA-13 for the statistical analyses and R version 4.1.3 [25] for data management.

We considered as exposures on a continuous scale the inflammatory markers (neutrophil, monocyte, and lymphocyte counts, and CRP), platelet parameters (platelet count, MPV, PDW), and allometric obesity indices (BMI, ABSI, and HI). For comparability, we calculated sex-specific z-scores (value minus mean, divided by standard deviation, SD) for all exposures, after log-transforming blood cell parameters and CRP to mitigate the right-skewness of their distributions. To examine associations between the exposures of interest, we calculated pairwise partial Pearson correlation coefficients adjusted for age at recruitment, separately in women and men.

We first considered each exposure individually and then combined all exposures in a joint, mutually adjusted model. We examined associations overall and heterogeneity according to sex and follow-up time (dichotomising at ≥ 6 years, which provided a comparable number of cases in the two groups). We derived hazard ratios (HRs) and 95% confidence intervals (CI) with delayed-entry Cox proportional hazards models, which account for left-truncation, as they are conditional on surviving cancer-free to recruitment. We interpreted the HRs per one SD increase of the exposures. The underlying time scale was age, with origin − the date of birth; entry time − the date at recruitment; exit time − the earliest of the date of diagnosis of the first primary incident cancer, or death, or last complete follow-up. For models examining kidney cancer cases with follow-up < 6 years, all participants were included but exit time was censored at 6 years after recruitment. For models examining kidney cancer cases with follow-up ≥ 6 years, entry time was lagged with 6 years and participants with follow-up < 6 years were excluded from the analysis. To select covariates, we examined the pairwise associations of a priory determined set of candidate covariates with the exposures and with kidney cancer risk (in participants overall adjusting for sex), and retained those covariates associated with at least one of the exposures and the outcome (Supplementary Figure S1). Candidate covariates included variables used and defined in our previous analyses examining associations of obesity indices with inflammatory markers and cancer [9, 18, 26, 27]. The final models for individual exposures were stratified by age at recruitment (five-year categories), smoking status and intensity (never smoked; just tried; former occasional; former regular quit ≥ 20 years; former regular quit ≥ 10 years; former regular quit < 10 years; current occasional; current regular ≤ 10 cigarettes/day; current regular > 10 cigarettes/day), and sex (except for sex-specific models), and were adjusted for height (sex-specific z-scores), alcohol consumption (≤ 3 times/month; ≤4 times/week; daily), physical activity (less active; moderately active; very active), education (primary school; secondary/vocational; university degree), diabetes (either self-reported or HbA1c ≥ 48mmol/mol), use of lipid lowering drugs, hypertension with or without antihypertensive treatment, use of antiaggregant/anticoagulants, and dietary intake of fruit and vegetables (> 3 portions/day fruit or > 5 portions/day vegetables), red and processed meat (> 2 times/week red meat or > once/week processed meat), and fibre (> 16 g/day). The final combined models included all exposures and were thus additionally adjusted for other exposures. We tested the proportional hazards assumption based on Schoenfeld residuals. We tested heterogeneity by sex (p_sex_) and follow-up time (p_follow−up_) with the augmentation method of Lunn and McNeil [28].

In a secondary analysis, we compared the associations of traditional body shape indices (waist circumference, WHR, hip circumference) with kidney cancer risk with the associations of the corresponding allometric body shape indices (ABSI, WHI, HI) with kidney cancer risk. We examined each obesity index individually as exposure in models fully stratified and adjusted for covariates and in models further adjusted for inflammatory markers and platelet parameters.

In sensitivity analyses, we examined the influence of covariates comparing the HR estimates from the final models for individual exposures (fully stratified and adjusted for covariates in the main dataset) with the HR estimates from unadjusted models stratified only by sex, models additionally stratified by smoking status and intensity, and models additionally adjusted for hypertension. We further compared the final models combining all exposures (fully stratified and adjusted for covariates in the main dataset) with models additionally adjusted for all remaining candidate covariates. To explore the influence of exclusions for missing exposures, we examined associations with individual exposures including all cohort participants with available measurement for a given exposure (extended dataset) in models stratified only by sex and in models fully stratified and adjusted for covariates. To explore the influence of exclusions for missing covariates, we examined associations in the complete-covariates dataset, using models combining all exposures (fully stratified and adjusted for covariates). To identify the most influential exposure in the mutual adjustment, we added sequentially other exposures to models for individual exposures (fully stratified and adjusted for covariates in the main dataset) up to the complete combined models. Last, we excluded from the main dataset cohort participants reporting use of immunosuppressants, as these can influence leucocyte function.

Missing values for covariates were below < 1% of the main analysis dataset (< 2% of the extended dataset), except 2.5% with missing number of cigarettes/day in current smokers. We replaced the limited number of missing values for covariates with the median category for each sex, as previously described [27]. Further details on missingness are included in the legend of Supplementary Table S2.

Tests of statistical significance were two-sided and were considered statistically significant at *p* < 0.05.

## Results

### Cohort characteristics

During a mean follow-up of 10.4 years, 1086 kidney cancer cases were ascertained (389 in women; 697 in men; 504 within 6 years of follow-up; 582 after 6 years) in 396,482 participants (211,843 women; 184,639 men; 372,186 with follow-up ≥ 6 years) (Supplementary Table S2). Women had lower BMI and ABSI and were less likely to have diabetes or hypertension or to use lipid-lowering drugs or antiaggregant/anticoagulants compared to men. Women were also less likely to smoke, to consume alcohol daily, or to be very physically active. Biomarkers differed little between women and men, except that platelet count was higher in women and monocyte count was higher in men. There was no material difference in participant characteristics for the extended and the complete-covariates datasets compared to the main study dataset (complete-exposures dataset).

**Fig. 1:**
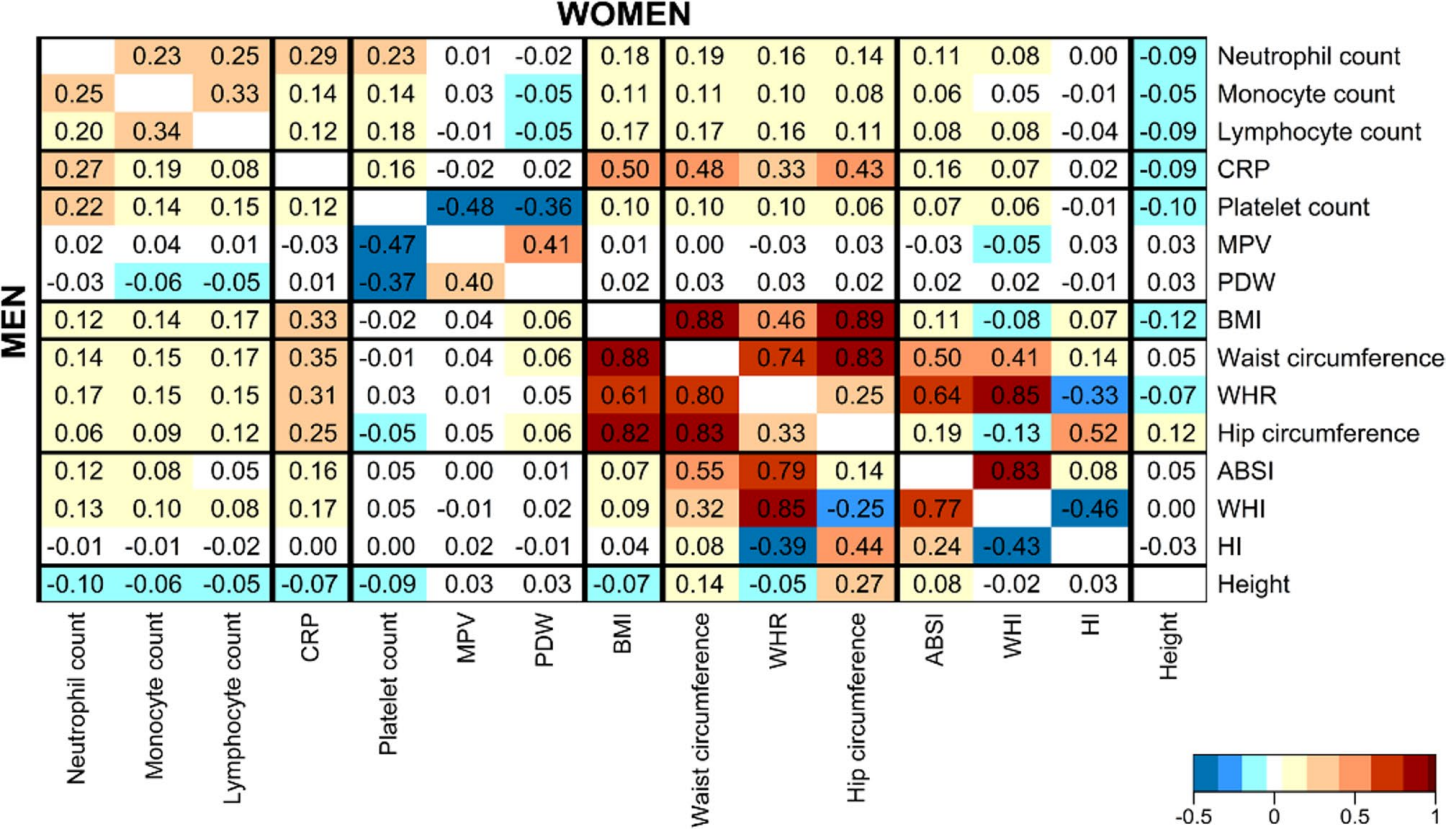
Pairwise correlations between inflammatory markers, platelet parameters, and obesity indices. ABSI– a body shape index; BMI– body mass index; CRP– C-reactive protein; HI– hip index; MPV– mean platelet volume; PDW– platelet distribution width; WHI– waist-to-hip index; WHR– waist-to-hip ratio. Partial Pearson correlation coefficients adjusted for age at recruitment were calculated for variables on a continuous scale (sex-specific z-scores, value minus mean divided by standard deviation, after log-transformation for biomarkers)

All inflammatory markers were positively correlated with each other and with platelet count, but most strongly monocyte with lymphocyte count, CRP with neutrophil count, and platelet with neutrophil count (Fig. 1). MPV and PDW were positively corelated only with each other and were negatively correlated with platelet count. BMI was positively correlated with all inflammatory markers, but most strongly with CRP, especially in women. The corresponding positive correlations with ABSI were weaker, while HI was not correlated with any of the biomarkers. Waist and hip circumferences were strongly positively correlated with each other and with BMI. WHR was positively correlated with BMI and hip circumference but, similarly to WHI, was negatively correlated with HI.

### Associations of inflammatory markers with kidney cancer risk

**Fig. 2:**
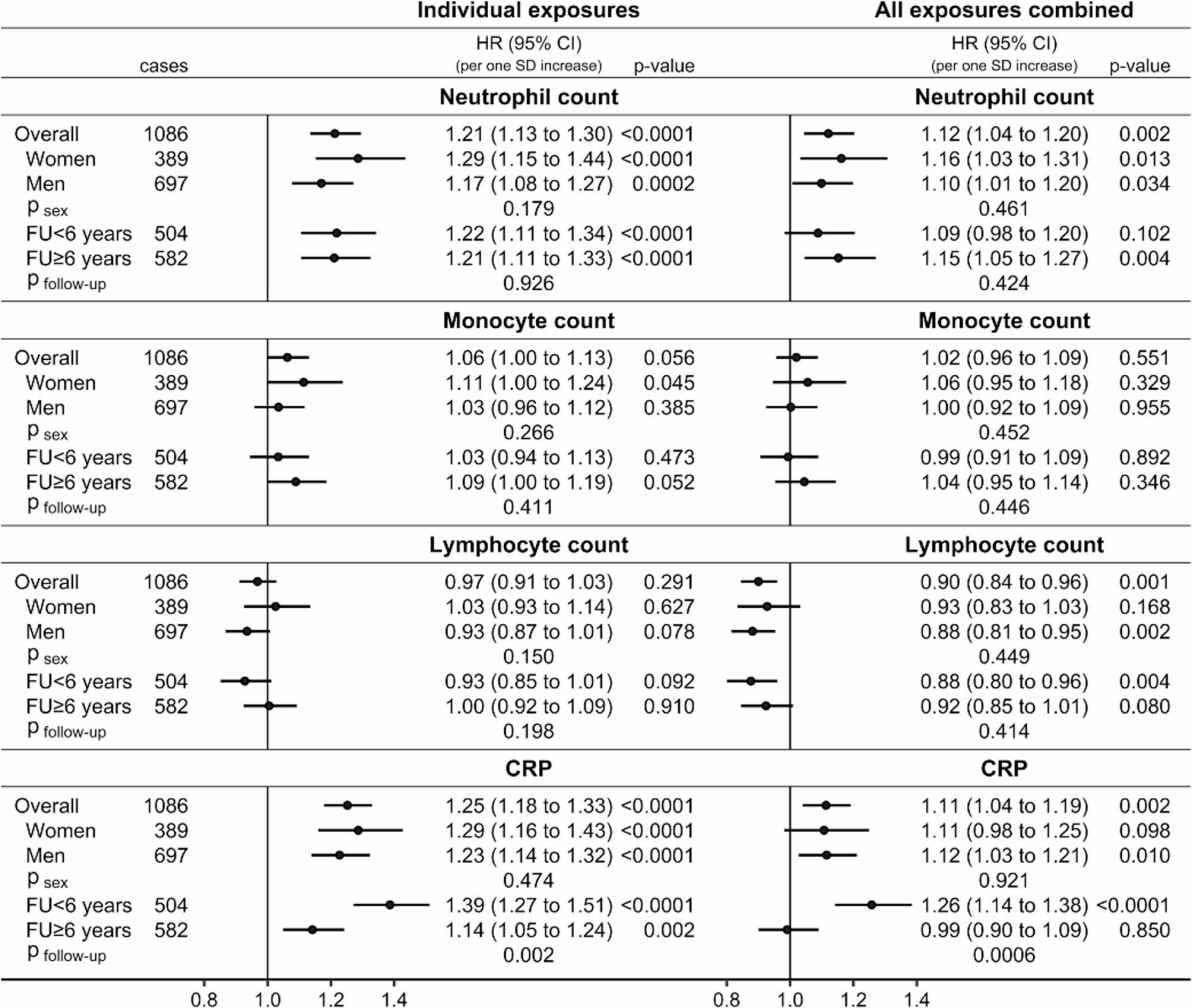
Associations of inflammatory markers with kidney cancer risk. CI– confidence interval; CRP– C-reactive protein; HR– hazard ratio; SD– standard deviation; cases– number of kidney cancer cases; p-value– Wald test for the individual term; p _sex_ / p _follow−up_– p-value for heterogeneity by sex or follow-up time obtained with the augmentation method of Lunn and McNeil [28]; FU < 6 years– cases diagnosed within the first 6 years of follow-up, censoring follow-up at 6 years; FU ≥ 6 years– cases diagnosed after 6 years of follow-up, lagging entry time with 6 years and excluding participants with follow-up < 6 years. Individual exposures– Cox proportional hazards models examining individually each inflammatory marker specified in the header (sex-specific z-scores, value minus mean divided by standard deviation after log-transformation), stratified by age at recruitment, smoking status and intensity, and sex (except for sex-specific models), and adjusted for height (sex-specific z-scores), alcohol consumption, physical activity, education, diabetes, use of lipid-lowering drugs, hypertension with or without treatment, use of antiaggregant/anticoagulants, and dietary intake of fruit and vegetables, red and processed meat, and fibre. All exposures combined– models fully stratified and adjusted for covariates, including all inflammatory markers (neutrophil, monocyte, and lymphocyte counts, and CRP) and additionally adjusted for platelet parameters (platelet count, mean platelet volume, platelet distribution width) and allometric obesity indices (body mass index, a body shape index, hip index)

Conditional on covariates, neutrophil count (HR = 1.21; 95%CI: 1.13 to 1.30 per one SD increase) and CRP (HR = 1.25; 95%CI: 1.18 to 1.33 per one SD increase) were positively associated with kidney cancer risk in participants overall, while monocyte count was most clearly positively associated in women (HR = 1.11; 95%CI: 1.00 to 1.24 per one SD increase) but there were no statistically significant associations with lymphocyte count (Fig. 2). There was no evidence for heterogeneity by sex or follow-up time, except that the positive association with CRP was stronger for shorter follow-up time (p_follow−up_=0.002). In the combined models, mutually adjusting inflammatory markers, platelet parameters, and obesity indices, the positive association with neutrophil count was partly attenuated (HR = 1.12; 95%CI: 1.04 to 1.20 in participants overall) but remained statistically significant for longer follow-up time, while the positive association with CRP was also partly attenuated (HR = 1.11; 95%CI: 1.04 to 1.19 in participants overall) but was observed only for shorter follow-up time (HR = 1.26; 95%CI: 1.14 to 1.38) and was abolished for longer follow-up time (HR = 0.99; 95%CI: 0.90 to 1.09; p_follow−up_=0.0006). The positive association with monocyte count was abolished in the combined models and an inverse association with lymphocyte count was revealed (HR = 0.90; 95%CI: 0.84 to 0.96 per one SD increase in participants overall), with no evidence for heterogeneity by sex or follow-up time.

### Associations of platelet parameters with kidney cancer risk

**Fig. 3:**
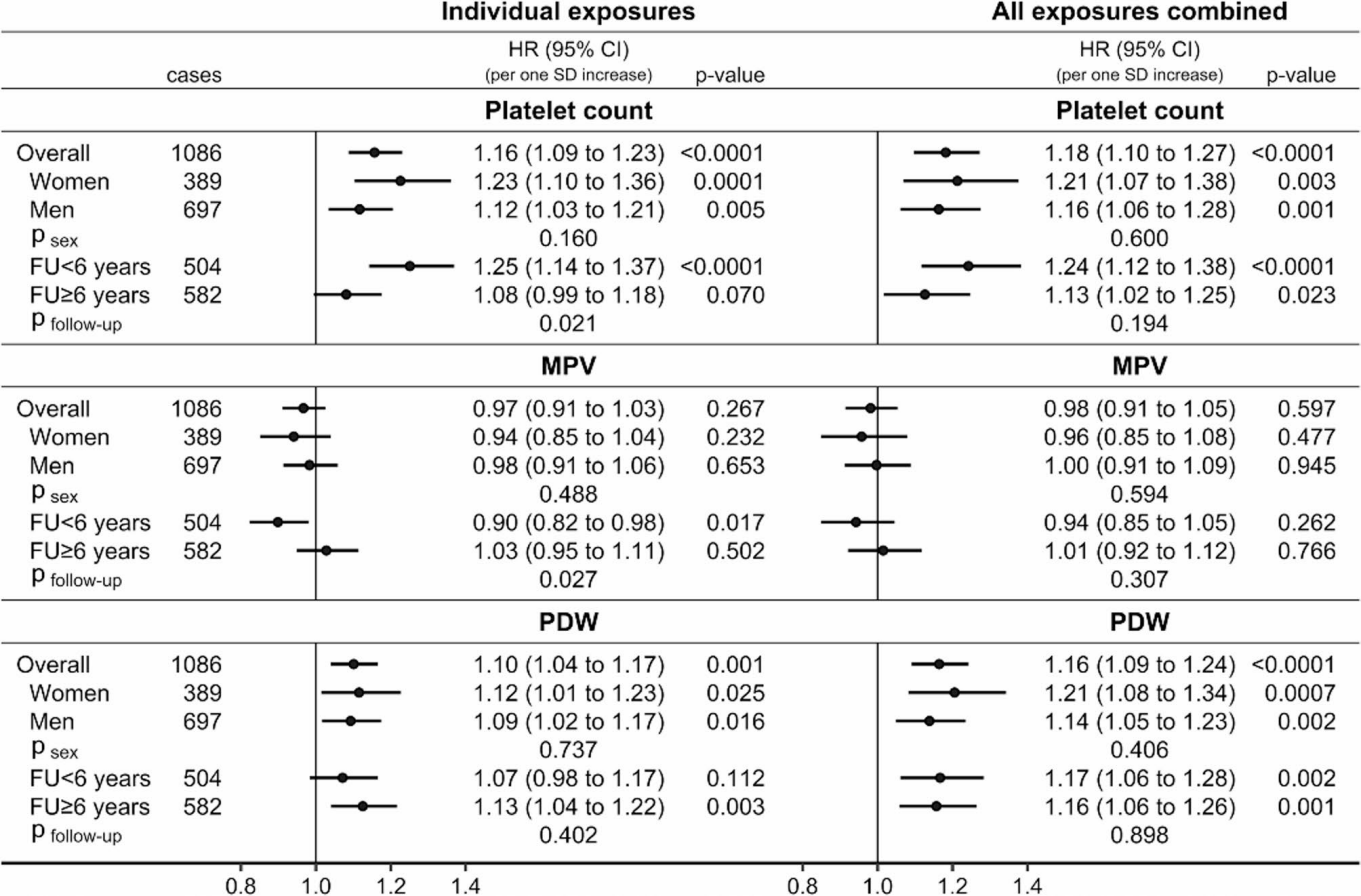
Associations of platelet parameters with kidney cancer risk. CI– confidence interval; HR– hazard ratio; MPV– mean platelet volume; PDW– platelet distribution width; SD– standard deviation; cases– number of kidney cancer cases; p-value– Wald test for the individual term; p _sex_ / p _follow−up_– p-value for heterogeneity by sex or follow-up time obtained with the augmentation method of Lunn and McNeil [28]; FU < 6 years– cases diagnosed within the first 6 years of follow-up, censoring follow-up at 6 years; FU ≥ 6 years– cases diagnosed after 6 years of follow-up, lagging entry time with 6 years and excluding participants with follow-up < 6 years. Individual exposures– Cox proportional hazards models examining individually each platelet parameter specified in the header (sex-specific z-scores, value minus mean divided by standard deviation after log-transformation), stratified by age at recruitment, smoking status and intensity, and sex (except for sex-specific models), and adjusted for height (sex-specific z-scores), alcohol consumption, physical activity, education, diabetes, use of lipid-lowering drugs, hypertension with or without treatment, use of antiaggregant/anticoagulants, and dietary intake of fruit and vegetables, red and processed meat, and fibre. All exposures combined– models fully stratified and adjusted for covariates, including all platelet parameters (platelet count, MPV, PDW) and additionally adjusted for inflammatory markers (neutrophil, monocyte, and lymphocyte counts, and C-reactive protein) and allometric obesity indices (body mass index, a body shape index, hip index)

Conditional on covariates, platelet count was positively associated with kidney cancer risk (HR = 1.16; 95%CI: 1.09 to 1.23 per one SD increase in participants overall), similarly in women and men, but more strongly for shorter follow-up time (p_follow−up_=0.021) (Fig. 3). MPV was not associated with kidney cancer risk in participants overall but was inversely associated for shorter follow-up time (HR = 0.90; 95%CI: 0.82 to 0.98 per one SD increase; p_follow−up_=0.027). PDW was positively associated with kidney cancer risk (HR = 1.10; 95%CI: 1.04 to 1.17 per one SD increase in participants overall), with no material difference by sex or follow-up time. Mutual adjustment of platelet parameters, inflammatory markers, and obesity indices minimally strengthened the positive association with platelet count (HR = 1.18; 95%CI: 1.10 to 1.27 in participants overall) but the difference by follow-up time was no longer statistically significant (HR = 1.24; 95%CI: 1.12 to 1.38 with follow-up < 6 years; HR = 1.13; 95%CI: 1.02 to 1.25 with follow-up ≥ 6 years; p_follow−up_=0.194). Mutual adjustment of all exposures abolished the isolated inverse association with MPV for shorter follow-up time and strengthened the positive association with PDW (HR = 1.16; 95%CI: 1.09 to 1.24 in participants overall).

### Associations of obesity indices with kidney cancer risk

**Fig. 4:**
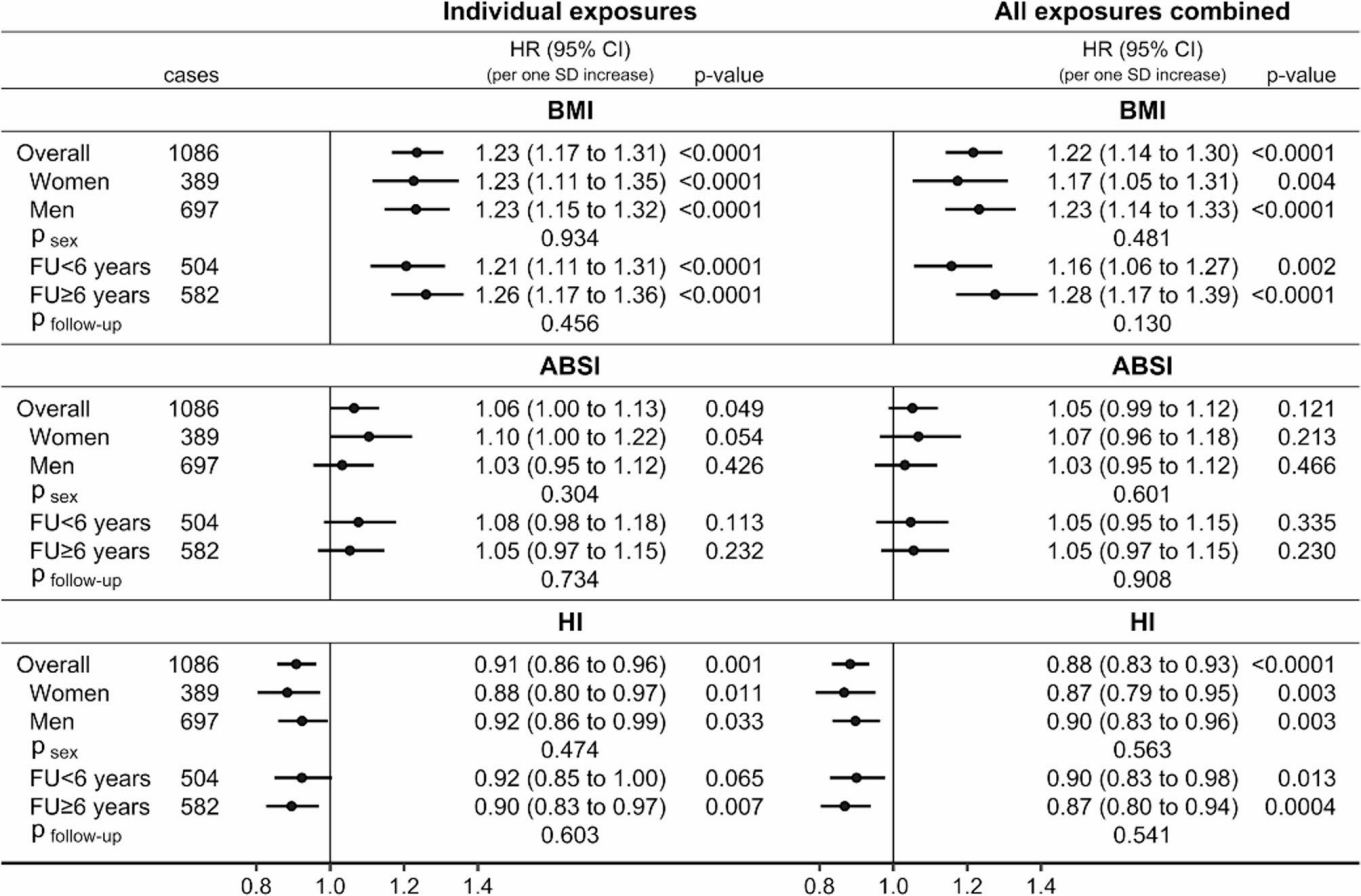
Associations of allometric obesity indices with kidney cancer risk. ABSI– a body shape index; BMI– body mass index; CI– confidence interval; HI– hip index; HR– hazard ratio; SD– standard deviation; cases– number of kidney cancer cases; p-value– Wald test for the individual term; p _sex_ / p _follow−up_– p-value for heterogeneity by sex or follow-up time obtained with the augmentation method of Lunn and McNeil [28]; FU < 6 years– cases diagnosed within the first 6 years of follow-up, censoring follow-up at 6 years; FU ≥ 6 years– cases diagnosed after 6 years of follow-up, lagging entry time with 6 years and excluding participants with follow-up < 6 years Individual exposures– Cox proportional hazards models examining individually each allometric obesity index specified in the header (sex-specific z-scores, value minus mean divided by standard deviation), stratified by age at recruitment, smoking status and intensity, and sex (except for sex-specific models), and adjusted for height (sex-specific z-scores), alcohol consumption, physical activity, education, diabetes, use of lipid-lowering drugs, hypertension with or without treatment, use of antiaggregant/anticoagulants, and dietary intake of fruit and vegetables, red and processed meat, and fibre. All exposures combined– models fully stratified and adjusted for covariates, including all allometric obesity indices (BMI, ABSI, and HI) and additionally adjusted for inflammatory markers (neutrophil, monocyte, and lymphocyte counts, and C-reactive protein) and platelet parameters (platelet count, mean platelet volume, platelet distribution width)

Conditional on covariates, BMI (HR = 1.23; 95%CI: 1.17 to 1.31 per one SD increase) and ABSI (HR = 1.06; 95%CI: 1.00 to 1.13 per one SD increase) were positively associated, while HI (HR = 0.91; 95%CI: 0.86 to 0.96 per one SD increase) was inversely associated with kidney cancer risk in participants overall (Fig. 4). There was no evidence for heterogeneity by sex or follow-up time. Mutual adjustment of obesity indices, inflammatory markers, and platelet parameters did not materially affect the positive association with BMI (HR = 1.22; 95%CI: 1.14 to 1.30) or the inverse association with HI (HR = 0.88; 95%CI: 0.83 to 0.93) in participants overall, but partly attenuated the associations with BMI in women and for shorter follow-up time and abolished the weak positive association of ABSI with kidney cancer risk.

**Fig. 5:**
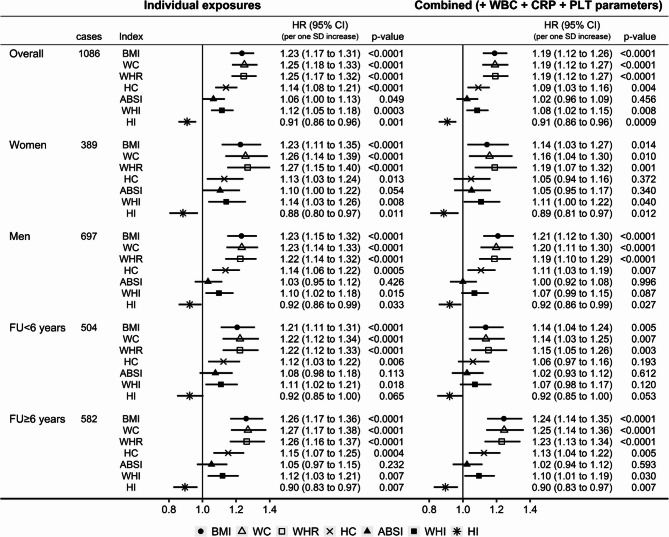
Comparison of traditional and allometric body shape indices. ABSI– a body shape index; BMI– body mass index; CI– confidence interval; CRP– C-reactive protein; HC– hip circumference; HI– hip index; HR– hazard ratio; PLT– platelet parameters; SD– standard deviation; WBC– white blood cell (leucocyte) counts; WC– waist circumference; WHI– waist-to-hip index; WHR– waist-to-hip ratio; cases– number of kidney cancer cases; p-value– Wald test for the individual term; FU < 6 years– cases diagnosed within the first 6 years of follow-up, censoring follow-up at 6 years; FU ≥ 6 years– cases diagnosed after 6 years of follow-up, lagging entry time with 6 years and excluding participants with follow-up < 6 years. Individual exposures– Cox proportional hazards models examining individually each obesity index specified in the header (sex-specific z-scores, value minus mean divided by standard deviation), stratified by age at recruitment, smoking status and intensity, and sex (except for sex-specific models), and adjusted for height (sex-specific z-scores), alcohol consumption, physical activity, education, diabetes, use of lipid-lowering drugs, hypertension with or without treatment, use of antiaggregant/anticoagulants, and dietary intake of fruit and vegetables, red and processed meat, and fibre. Combined– models fully stratified and adjusted for covariates, including each obesity index individually with additional adjustment for inflammatory markers (neutrophil, monocyte, and lymphocyte counts, and C-reactive protein) and platelet parameters (platelet count, mean platelet volume, platelet distribution width)

Conditional on covariates, all three traditional body shape indices were positively associated with kidney cancer risk, with comparable associations to BMI for waist circumference (HR = 1.25; 95%CI: 1.18 to 1.33 per one SD increase) and WHR (HR = 1.25; 95%CI: 1.17 to 1.32 per one SD increase) but a weaker association for hip circumference (HR = 1.14; 95%CI: 1.08 to 1.21 per one SD increase) (Fig. 5). Adjustment for inflammatory markers and platelet parameters partly attenuated the associations of waist circumference and WHR and abolished the positive association of hip circumference with kidney cancer risk in women and for shorter follow-up time. WHI was associated positively with kidney cancer risk when examined individually (HR = 1.12; 95%CI: 1.05 to 1.18 per one SD increase in participants overall) and after adjustment for inflammatory markers and platelet parameters (HR = 1.08; 95%CI: 1.02 to 1.15), unlike ABSI.

### Sensitivity analyses

In unadjusted models, the positive associations with neutrophil and monocyte counts, CRP, BMI, and ABSI with kidney cancer risk were slightly stronger and there was some evidence for a positive association of lymphocyte count with kidney cancer risk, more specifically in women and for longer follow-up time (Supplementary Figure S2). Adjustment for smoking and hypertension accounted for most of the attenuation of the positive associations of inflammatory markers and ABSI with kidney cancer risk observed in the models fully adjusted for covariates, with no material influence of the additional adjustment for other covariates. Adjustment for smoking, however, did not influence the positive association of BMI with kidney cancer risk and this was attenuated mainly after adjustment for hypertension, while the adjustment for the remaining covariates contributed to a small additional attenuation mainly in women. Adjustment for covariates influenced little the positive associations of platelet parameters and the inverse association of HI with kidney cancer risk. Association estimates based on the extended dataset, using all available measurements for each individual exposure, did not differ materially from estimates based on the main complete-exposures dataset (Supplementary Figure S2).

The sequential addition of exposures for mutual adjustment showed that adjustment for CRP mainly accounted for the attenuation of the positive association of neutrophil count with kidney cancer risk in the models combining all exposures and adjustment for neutrophil count accounted for the abolition of the positive association with monocyte count, while adjustment for neutrophil count and BMI accounted for the attenuation of the positive association with CRP and the shift towards the inverse of the association with lymphocyte count (Supplementary Figure S3). The positive association with platelet count was stronger after adjustment for PDW but was restored after further adjustment for neutrophil count. Adjustment for platelet count accounted for the stronger positive association of PDW with kidney cancer risk and the abolition of the isolated inverse association with MPV for shorter follow-up time in the combined models. Mainly adjustment for CRP accounted for the attenuation of the positive associations with BMI and ABSI.

There were no material differences of association estimates compared to the main models when examining all primary kidney cancers (including second and subsequent, following a primary cancer in other location), after excluding cohort participants with self-reported use of immunosuppressants, after excluding participants with missing any covariate (complete-covariates dataset), or after additional adjustment of the models combining all exposures for all candidate covariates (Supplementary Figure S4).

## Discussion

In this study, conditional on each other and covariates, neutrophil and platelet counts, and PDW, as well as BMI and CRP, were associated positively, while lymphocyte count and HI were associated inversely with kidney cancer risk. There were no major sex differences, but the association with CRP was stronger for shorter follow-up time.

Our findings corroborate previous reports in the UK Biobank cohort based on a shorter follow-up time (on average 7 years) and hence with a stronger influence of the cases diagnosed within the first 6 years post recruitment [11, 13, 18]. They are also directionally consistent with the previously reported associations with ratios based on leucocyte and platelet counts [13]. The positive association of neutrophil count with kidney cancer risk is further compatible with the generally expected increase in the number of circulating neutrophils with cancer progression and their association with a poorer prognosis [29], as well as with the poor kidney cancer prognosis associated with higher tumour infiltration with neutrophils [30]. We did not observe, however, a stronger positive association with neutrophil count for shorter compared to longer follow-up time. The inverse association with lymphocyte count is compatible with the shorter survival in kidney cancer patients with low lymphocyte count at diagnosis [31]. The positive association with CRP we observed for shorter follow-up time corresponds to the earlier report for UK Biobank [11] and is compatible with high CRP at diagnosis being a poor prognostic marker [32]. We have additionally shown, however, that there are no sex differences in this association and that it was lost for longer follow-up time after accounting for neutrophil count and BMI.

Our findings are also in agreement with the previously reported stronger positive association of platelet count for shorter follow-up time [16], but we have additionally shown that there were no major sex differences in this association. The inverse prospective association with MPV that we observed for shorter follow-up time is compatible with the previously reported lower MPV in kidney cancer patients at diagnosis [17]. This association, however, was lost when adjusting for platelet count and thus may reflect a cancer-induced generation of mature platelets. This would correspond to the inverse correlation between MPV and platelet count and the fact that the larger barbell-shaped proplatelet intermediates undergo fission in the process of maturation to form two smaller size mature platelets [33]. Although PDW is also associated inversely with platelet count, our prospective analysis has shown a positive association of PDW with kidney cancer risk, stronger after conditioning on platelet count, which is compatible with the fact that variable platelet size as reflected in PDW is considered a marker of platelet activation [34].

We can thus propose that an immune-cell and inflammation-related mechanism is operational in kidney cancer development separately from obesity and has two aspects. Prospectively, a long-acting pathway involves neutrophils as representatives of the innate immune system and is assisted by activated platelets (as indicated by the prospective positive associations with neutrophil and platelet counts, and PDW). Closer to kidney cancer diagnosis, a tumour-induced thrombo-inflammation develops, which likely involves cytokine release and switches a positive feed-back loop of stimulated thrombopoiesis (as indicated by stronger positive associations with CRP and PLT and an inverse association with MPV for shorter follow-up time). A suppression of the anti-tumour immune response appears relevant throughout kidney cancer development, as indicated by an inverse association with lymphocyte count, which was influenced little by follow-up time.

There are ample mechanistic studies examining the crosstalk of leucocytes and platelets with developing cancers. During kidney cancer development, cancer cells release cytokines to attract infiltrating immune-inhibitory cells, which block the anti-tumour immune response [35]. Corresponding to the cancer-related inflammation, inflammatory cytokines are higher at kidney cancer diagnosis [36], although CRP itself is unlikely to have a causal role, as there was little evidence for association of genetically-predicted CRP with kidney cancer risk [11]. In conditions of cancer-related inflammation, neutrophils − the largest leucocyte subset in peripheral blood, change functionality, infiltrate the tumour site, interact with cancer cells and other infiltrating leucocytes, and facilitate cancer development and progression [37, 38]. Although circulating neutrophils in kidney cancer patients show some differences compared to healthy controls (immature phenotype with higher metabolic activity), the tumour infiltrating neutrophils themselves exhibit an immunosuppressive phenotype and correlate positively with BMI and subcutaneous adipose tissue [39]. Tumour associated neutrophils have downregulated cytotoxicity-related genes and up-regulated cytokine-related genes producing chemoattractants for all types of leucocytes [40]. The type of cytokine produced in the tumour microenvironment determines whether infiltrating neutrophils would develop pro-oncogenic or anti-oncogenic properties [41, 42]. Blood-derived monocytes are also attracted by tumour-released cytokines and give rise to tumour infiltrating macrophages associated with poor kidney cancer prognosis [43], although we did not observe a corresponding prospective positive association with monocyte count conditional on neutrophil count. Myeloid cells (neutrophils and monocytes) constitute only one third of the infiltrating immune cells within the kidney cancer microenvironment, while half of all infiltrating immune cells are CD4 and CD8 T-cell lymphocytes [44]. T-cells aid tumor detection and destruction and are important for immunosurveillance, but their depletion or suppression induced by the developing tumour contributes to evasion of the anti-tumour immune response and poor prognosis [45]. Natural killer (NK) cells are another lymphocyte subtype associated with immune surveillance and tumour destruction and in kidney cancer patients, peripheral and infiltrating NK cells differ, with the latter showing higher expression of genes related to angiogenesis and immunosuppression [46]. Compared to other cancers, however, kidney cancers have considerably higher proportion of infiltrating NK cells [47]. Notably, a higher proportion of NK cells has been observed in less advanced kidney cancers [47] and has been associated with higher cytotoxicity [48] and better survival [49].

Further involved in the cancer driven inflammation are activated platelets, which release pro-angiogenic factors stimulating tumour angiogenesis and pro-inflammatory cytokines attracting immune cells towards the tumour site [50]. Platelets facilitate cancer progression and metastases by inducing deep vein thrombosis and formation of neutrophil extracellular traps, which protect cancer cells [15]. Platelets promote evasion of the adaptive immune response by increasing the expression in kidney cancer cell lines of immune checkpoint proteins [51], which are associated with poor kidney cancer prognosis [52]. Thus, all the above-described cancer-related interactions of inflammatory cytokines, neutrophils, lymphocytes, and platelets, could explain the associations with kidney cancer risk that we observed for shorter follow-up time. Less clear is, however, whether neutrophils can act in the absence of cancer cells to facilitate carcinogenesis as opposed to cancer progression or whether kidney cancers have a very long latent period, which could explain the longer-term prospective associations with neutrophil and platelet counts, and PDW.

BMI, notably, retained a longer-term positive association with kidney cancer risk conditional on inflammatory markers and platelet parameters, while the weak positive association with the allometric index of abdominal obesity ABSI was abolished after adjustment for inflammatory markers. A positive association with ABSI has previously been reported in post-menopausal women from the Women’s Health Initiative [53] and in an unadjusted model including UK Biobank women and men combined, but the later was lost after adjustment for smoking [54]. Although insulin resistance is one of the mechanisms by which obesity can be implicated in cancer development [7] and both BMI and ABSI were positively associated with glycated haemoglobin in UK Biobank [9], our current analysis lends little support for a contribution of obesity-related insulin resistance to kidney cancer development, since the positive association with abdominal obesity reflected in ABSI could not be retained after adjustment for covariates (including diabetes), inflammatory markers and platelet parameters. Although waist circumference and WHR were positively associated with kidney cancer risk, in agreement with previous reports [3, 55], our study highlights the caveats of using traditional body shape indices of abdominal obesity. In the light of the limited evidence of association of ABSI with kidney cancer risk, the positive association of waist circumference with kidney cancer risk would be explained by the strong positive correlation of waist circumference with BMI, which is factored out by design in ABSI. The positive association of WHR with kidney cancer risk would similarly be explained in part by the positive correlation of WHR with BMI. The inverse association of hip size with kidney cancer risk, however, would also have a contribution, as WHI, in which the positive correlation of WHR with BMI is factored out by design, remained positively associated with kidney cancer risk after adjustment for inflammatory markers and platelet parameters, and both WHR and WHI were negatively correlated with HI, in which the strong positive correlation of hip circumference with BMI is factored out by design.

A BMI-related mechanism more specific to general than to abdominal obesity could be peripherally derived oestrogens, which are higher in men and post-menopausal women with obesity [56]. This would be compatible with an animal model paradigm of kidney cancer induction by oestradiol and 4-hydroxy-oestradiol in the Syrian hamster, which is suppressed by anti-oestrogens (tamoxifen) and hence requires an oestrogen receptor [57, 58]. Circulating oestradiol levels, however, are hard to measure and were undetectable in the larger part of UK Biobank men and post-menopausal women [56]. Correspondingly, a previous study found no evidence of association of circulating oestradiol with kidney cancer risk considering only UK Biobank participants with detectable oestradiol [59]. Another pathway more relevant to general than to abdominal obesity could be adipokine production [7]. Although leptin produced by local adipose tissue near the tumour does not affect proliferation, it reduces cell adhesion and enhances the migration and invasiveness of renal epithelial cells [60].

The consistent inverse association of HI with kidney cancer risk, which remained after conditioning on inflammatory markers and platelet parameters in our study, is intriguing. Although large hip size is associated with higher circulating oestradiol [56] and oestrogen production is higher in gluteofemoral adipose tissue [61], an oestrogen-dependent mechanism could not explain an inverse association with HI, since oestrogens induce kidney cancer in animal models [57, 58]. Nevertheless, large hip size is a feminine feature [62] and the unknown factors contributing to an inverse association of HI with kidney cancer risk could be related to the distinctly lower kidney cancer incidence in women compared to men [1].

Major strengths of our study are the prospective cohort design with a substantial number of incident kidney cancer cases, the availability of haematological measurements and detailed information for smoking intensity and duration of smoking cessation and for other major lifestyle factors, which permitted adjustment for confounding. Clear limitations of our study are the lack of information about lymphocyte subtypes, or leucocyte and platelet functionality, as well as about immune cell or adipose tissue infiltration in the kidney. We did not have information on inflammatory cytokines, adipokines, or blood coagulation factors either. We also had to assume that the baseline levels of the exposures remained constant during cancer follow-up, as a second biomarker measurement was available only for less than 5% of study participants. Further, UK Biobank includes mainly participants with white ethnic background, so there was no adequate information to examine other ethnicities. UK Biobank participants also have a healthier lifestyle compared to the general population, which may have limited variability in the exposures [63]. Last, although we have examined prospective associations, further mechanistic investigations would be required to demonstrate causality.

## Conclusions

Prospective positive associations of neutrophil and platelet counts, PDW, and BMI, conditional on each other and covariates, with kidney cancer risk support longer-acting pathways with an immune-cell-related component, likely involving a collaboration of neutrophils and activated platelets, and a separate component related to general rather than abdominal obesity. Stronger positive associations of CRP and platelet count and an accompanying inverse association of MPV with kidney cancer risk for shorter follow-up time support a cancer-induced thrombo-inflammation with a likely involvement of inflammatory cytokine synthesis and a stimulated production of smaller mature platelets. An inverse association of lymphocyte count with kidney cancer risk is compatible with a suppression of anti-tumour responses during kidney cancer development and progression. An inverse association of hip size reflected in HI with kidney cancer risk has at present an unknown underlying mechanism. The described associations were similar in women and men.

## Supplementary Information

Below is the link to the electronic supplementary material.

Supplementary Material 1

## Data Availability

The dataset analysed in the current study was used under license and cannot be made freely available in a public repository or obtained from the authors due to restrictions related to privacy regulations and informed consent of the participants. Access to the data, however, can be obtained by bona fide researchers from UK Biobank, subject to approval of the research project and a material transfer agreement. For information on how to gain access to UK Biobank data, please follow the instructions at https://www.ukbiobank.ac.uk/enable-your-research Further queries related to the data could be addressed to the corresponding author Dr Sofia Christakoudi s.christakoudi@imperial.ac.uk.

## Abbreviations

ABSI: a body shape index
BMI: body mass index
CI: confidence interval
CRP: C-reactive protein
HI: hip index
HR: hazard ratio
ICD10: 10^th^ revision of the International Statistical Classification of Diseases
MPV: mean platelet volume
PDW: platelet distribution width
SD: standard deviation
UK: United Kingdom
WHI: waist-to-hip index
WHR: waist-to-hip ratio
