# Supplementary material for: Associations of leucocyte subtypes and platelet parameters with kidney cancer risk in the UK Biobank cohort
Source: J Inflamm (Lond). 2025 Aug 11;22:31. doi: 10.1186/s12950-025-00458-6 (PMC12337549; doi:10.1186/s12950-025-00458-6)
Supplement: Supplementary file 1 — Supplementary Material 1 [file 12950_2025_458_MOESM1_ESM.pdf]

Associations of leucocyte subtypes and platelet parameters with kidney cancer risk in the UK Biobank cohort

Sofia Christakoudi, Konstantinos K. Tsilidis, Marc J. Gunter, Elio Riboli

Supplementary Tables

Supplementary Table S1 Flow chart of study participants 2

Supplementary Table S2A Characteristics of study participants by sex 3

Supplementary Table S2B Characteristics of study participants by follow-up time 4

Supplementary Figures

Supplementary Figure S1 Pairwise associations of candidate covariates with the examined exposures and kidney cancer risk 6

Supplementary Figure S2A Sequential adjustment for covariates − associations of leucocyte counts and CRP with kidney cancer risk 8

Supplementary Figure S2B Sequential adjustment for covariates − associations of platelet parameters and BMI with kidney cancer risk 9

Supplementary Figure S2C Sequential adjustment for covariates – associations of allometric body shape indices with kidney cancer risk 10

Supplementary Figure S3A Sequential addition of exposures – associations of leucocyte counts and CRP with kidney cancer risk 12

Supplementary Figure S3B Sequential addition of exposures – associations of platelet parameters and allometric obesity indices with kidney cancer risk 13

Supplementary Figure S4A Additional sensitivity analyses – associations of leucocyte counts and platelet parameters with kidney cancer risk 15

Supplementary Figure S4B Additional sensitivity analyses – associations of CRP and allometric obesity indices with kidney cancer risk 16

References 18

Supplementary Table S1 Flow chart of study participants

	Exclusions	Overall	Women	Men
	Total (excluding withdrawals up to the analysis):	502,369	273,301	229,068
1.	Ethnic background (restricted to self-reported white) [a]	29,797	15,935	13,862
2.	Prevalent cancer at recruitment [a]	34,775	22,385	12,390
3.	Genetic & self-reported sex mismatch, or sex chromosome aneuploidy, or pregnant at recruitment [a]	842	469	373
4.	Antihemorrhagic agents [b]	604	590	14
	Total excluded except missing exposures	66,018 (13.1 %)	39,379 (14.4 %)	26,639 (11.6 %)
	**Total included (extended dataset)**	**436,351**	**233,922**	**202,429**
5.	Anthropometric measurements missing [a]	2034	978	1056
6.	Leucocyte and platelet measurements missing [c]	19,807	11,532	8275
7.	C-reactive protein measurements missing [d]	18,028	9569	8459
	Total excluded with missing exposures	39,869 (7.9 %)	22,079 (8.1 %)	17,790 (7.8 %)
	Total excluded overall (% from available)	105,887 (21.1 %)	61,458 (22.5 %)	44,429 (19.4 %)
	**Total included (main complete-exposures dataset)**	**396,482**	**211,843**	**184,639**
	Missing any covariate (% from main dataset)	13,985 (3.5 %)	6454 (3.0 %)	7531 (4.1 %)
	**Total included (complete-covariates dataset)**	**382,497**	**205,389**	**177,108**

The exclusion criteria were applied sequentially in the displayed order, counting each excluded cohort participant only once.

[a] – for UK Biobank Field names, definition of variables, and definition of prevalent cancer cases see Supplementary Methods in reference [18]. Note that the current selection includes the complete range of body mass index.

[b] – self-reported use of medications from Fields [20003-0/47] "Treatment/ medication code" with the following codes: 1140861766 (ethamsylate), 1140861832 (tranexamic acid), 1140861834 (cyklokapron 500mg tablet).

[c] – values were considered missing when no measurement was available. Zero values were replaced with half the lowest detected level (min=0.01*10^9/L) for neutrophil count (n=5 participants), monocyte count (n=123 main; n=127 extended), and lymphocyte count (n=5 main; n=6 extended).

[d] – C-reactive protein measurements in UK Biobank are marked with the following reportability codes:

1: "Reportable at assay and after aliquot correction, if attempted"
2: "Reportable at assay but not reportable after any corrections (too low)"
3: "Reportable at assay but not reportable after any corrections (too high)"
4: "Not reportable at assay (too low)"
5: "Not reportable at assay (too high)"

Values with code 1 were used as provided. Values with codes 2 and 4 were replaced with half the lowest detected level (0.08 mg/L) for 98 (main) / 101 (extended) participants. Values with codes 3 and 5 were replaced with the highest detected level (79.96 mg/L) for 233 / 243 participants.

Supplementary Table S2A Characteristics of study participants by sex

Sex	WOMEN			MEN		
**Dataset**	**Main**	**Extended**	**Complete**	**Main**	**Extended**	**Complete**
Cohort: n (%)	211,843 (53.4)	233,922 (53.6)	205,389 (53.7)	184,639 (46.6)	202,429 (46.4)	177,108 (46.3)
Cases: n (rate)	389 (175)	421 (171)	382 (177)	697 (367)	768 (369)	660 (362)
Age recruit (years) [a]	56.8 (8.0)	56.8 (8.0)	56.8 (8.0)	57.2 (8.1)	57.2 (8.1)	57.2 (8.1)
**BMI category [b]**						
<25 kg/m^2	85,696 (40.5)	93,780 (40.1)	83,640 (40.7)	46,218 (25.0)	50,434 (24.9)	44537 (25.1)
25 to <30 kg/m^2	77,806 (36.7)	85,527 (36.6)	75,384 (36.7)	91,616 (49.6)	99,572 (49.2)	88145 (49.8)
≥30 kg/m^2	48,341 (22.8)	53,682 (22.9)	46,365 (22.6)	46,805 (25.3)	51,402 (25.4)	44426 (25.1)
**Anthropometry [a]**						
BMI (kg/m^2)	27.0 (5.1)	27.0 (5.2)	26.9 (5.1)	27.8 (4.2)	27.9 (4.2)	27.8 (4.2)
ABSI	73.8 (5.0)	73.8 (5.0)	73.8 (4.9)	79.8 (4.1)	79.8 (4.1)	79.7 (4.1)
WHI	3.59 (0.27)	3.59 (0.27)	3.59 (0.27)	4.08 (0.23)	4.08 (0.23)	4.08 (0.22)
HI	64.3 (2.5)	64.3 (2.5)	64.3 (2.5)	49.1 (1.7)	49.1 (1.7)	49.1 (1.7)
WC (cm)	84.4 (12.4)	84.5 (12.5)	84.3 (12.4)	96.9 (11.3)	97.0 (11.3)	96.9 (11.2)
WHR	0.82 (0.07)	0.82 (0.07)	0.82 (0.07)	0.94 (0.06)	0.94 (0.07)	0.93 (0.06)
HC (cm)	103.2 (10.3)	103.3 (10.3)	103.2 (10.2)	103.5 (7.5)	103.5 (7.6)	103.5 (7.5)
Height (m)	162.6 (6.2)	162.6 (6.2)	162.7 (6.2)	175.9 (6.8)	175.9 (6.8)	175.9 (6.7)
**Diabetes [b]**						
Yes	7421 (3.5)	8377 (3.6)	7078 (3.4)	13,443 (7.3)	14,851 (7.3)	12,713 (7.2)
**Medications [b]**						
Lipid-lowering	26,639 (12.6)	29,570 (12.6)	25,530 (12.4)	43,093 (23.3)	47,277 (23.4)	41,255 (23.3)
Antiaggregants	22,864 (10.8)	25,261 (10.8)	22,086 (10.8)	38,605 (20.9)	42,437 (21.0)	37,055 (20.9)
**Hypertension [b]**						
No	159,916 (75.5)	176,349 (75.4)	155,448 (75.7)	123,847 (67.1)	135,693 (67.0)	119,045 (67.2)
Untreated	16,100 (7.6)	18,130 (7.8)	15,434 (7.5)	16,396 (8.9)	18,343 (9.1)	15,523 (8.8)
Treated	35,827 (16.9)	39,443 (16.9)	34,507 (16.8)	44,396 (24.0)	48,393 (23.9)	42,540 (24.0)
**Smoking status [b]**						
Never smoked	125,294 (59.1)	138,279 (59.1)	121,591 (59.2)	89,726 (48.6)	98,274 (48.5)	87,136 (49.2)
Former smoker	67,850 (32.0)	74,781 (32.0)	65,925 (32.1)	72,340 (39.2)	79,191 (39.1)	69,329 (39.1)
Current smoker	18,699 (8.8)	20,862 (8.9)	17,873 (8.7)	22,573 (12.2)	24,964 (12.3)	20,643 (11.7)
**Alcohol [b]**						
≤3 times/month	74,536 (35.2)	82,721 (35.4)	71,572 (34.8)	37,716 (20.4)	41,599 (20.5)	35,653 (20.1)
≤4 times/week	101,744 (48.0)	112,132 (47.9)	99,011 (48.2)	98,671 (53.4)	107,963 (53.3)	94,859 (53.6)
Daily	35,563 (16.8)	39,069 (16.7)	34,806 (16.9)	48,252 (26.1)	52,867 (26.1)	46,596 (26.3)
**Physical activity [b]**						
Less active	35,514 (16.8)	39,538 (16.9)	34,391 (16.7)	28,105 (15.2)	31,197 (15.4)	26,924 (15.2)
Moderately active	111,146 (52.5)	122,626 (52.4)	107,486 (52.3)	83,257 (45.1)	91,327 (45.1)	79,719 (45.0)
Very active	65,183 (30.8)	71,758 (30.7)	63,512 (30.9)	73,277 (39.7)	79,905 (39.5)	70,465 (39.8)
**Education [b]**						
Primary	35,488 (16.8)	39,132 (16.7)	33,836 (16.5)	31,442 (17.0)	34,398 (17.0)	29,335 (16.6)
Second/Vocational	110,410 (52.1)	122,583 (52.4)	106,537 (51.9)	91,357 (49.5)	100,866 (49.8)	87,113 (49.2)
University degree	65,945 (31.1)	72,207 (30.9)	65,016 (31.7)	61,840 (33.5)	67,165 (33.2)	60,660 (34.3)
**Dietary intake [c]**						
Fruit | Vegetables	115,024 (54.3)	126,875 (54.2)	111,775 (54.4)	79,135 (42.9)	86,602 (42.8)	76,145 (43.0)
Red | Process meat	87,744 (41.4)	96,954 (41.4)	84,930 (41.4)	113,030 (61.2)	123,987 (61.2)	108,302 (61.2)
Fibre (>16 g/day)	74,981 (35.4)	82,470 (35.3)	73,059 (35.6)	59,858 (32.4)	65,235 (32.2)	57,848 (32.7)
**Biomarkers [d]**						
PLT (*10^9/L)	260 (165−409)	260 (164−410)	260 (165−409)	232 (146−370)	232 (145−371)	232 (146−370)
MPV (fL)	9.3 (7.5−11.6)	9.3 (7.5−11.6)	9.3 (7.5−11.6)	9.2 (7.4−11.5)	9.2 (7.4−11.5)	9.2 (7.4−11.5)
PDW (%)	16.4 (15.5−17.4)	16.4 (15.5−17.4)	16.4 (15.5−17.4)	16.6 (15.6−17.6)	16.6 (15.6−17.6)	16.6 (15.6−17.6)
NEU (*10^9/L)	4.0 (2.1−7.6)	4.0 (2.1−7.6)	4.0 (2.1−7.6)	4.1 (2.1−7.8)	4.1 (2.1−7.8)	4.0 (2.1−7.8)
MO (*10^9/L)	0.41 (0.20−0.86)	0.41 (0.20−0.87)	0.41 (0.20−0.86)	0.49 (0.24−0.99)	0.49 (0.24−0.99)	0.49 (0.24−0.99)
LY (*10^9/L)	1.9 (1.0−3.5)	1.9 (1.0−3.5)	1.9 (1.0−3.5)	1.8 (1.0−3.3)	1.8 (1.0−3.3)	1.8 (1.0−3.3)
CRP (mg/L)	1.4 (0.2−11.9)	1.4 (0.2−12.0)	1.4 (0.2−11.8)	1.3 (0.2−9.9)	1.3 (0.2−10.0)	1.3 (0.2−9.8)

Supplementary Table S2B Characteristics of study participants by follow-up time

Follow-up time		FU<6 years [#]			FU≥6 years	
Dataset	Main	Extended	Complete	Main	Extended	Complete
Cohort: n (%)	396,482	436,351 (51.6)	382,497 (51.6)	372,186 (93.9)	409,425 (48.4)	359,330 (48.4)
Cases: n (rate)	504 (218)	550 (216)	482 (216)	582 (321)	639 (320)	560 (320)
Age recruit (years) [a]	57.0 (8.0)	57.0 (8.0)	57.0 (8.0)	56.8 (8.0)	56.8 (8.0)	56.7 (8.0)
**BMI category [b]**						
<25 kg/m^2	131,914 (33.3)	144,214 (33.0)	128,177 (33.5)	124,732 (33.5)	136,342 (33.3)	121,290 (33.8)
25 to <30 kg/m^2	169,422 (42.7)	185,099 (42.4)	163,529 (42.8)	158,828 (42.7)	173,476 (42.4)	153,391 (42.7)
≥30 kg/m^2	95,146 (24.0)	105,084 (24.1)	90,791 (23.7)	88,626 (23.8)	97,873 (23.9)	84,649 (23.6)
**Anthropometry [a]**						
BMI (kg/m^2)	27.4 (4.7)	27.4 (4.8)	27.3 (4.7)	27.4 (4.7)	27.4 (4.8)	27.3 (4.7)
ABSI	76.6 (5.5)	76.6 (5.5)	76.5 (5.5)	76.5 (5.4)	76.5 (5.4)	76.5 (5.4)
WHI	3.82 (0.35)	3.82 (0.35)	3.82 (0.35)	3.81 (0.35)	3.81 (0.35)	3.81 (0.35)
HI	57.2 (7.9)	57.2 (7.9)	57.2 (7.9)	57.3 (7.9)	57.3 (7.9)	57.3 (7.8)
WC (cm)	90.2 (13.5)	90.3 (13.5)	90.1 (13.4)	90.1 (13.4)	90.1 (13.5)	89.9 (13.4)
WHR	0.87 (0.09)	0.87 (0.09)	0.87 (0.09)	0.87 (0.09)	0.87 (0.09)	0.87 (0.09)
HC (cm)	103.4 (9.1)	103.4 (9.2)	103.3 (9.1)	103.3 (9.1)	103.4 (9.1)	103.3 (9.0)
Height (m)	168.8 (9.3)	168.8 (9.3)	168.8 (9.2)	168.8 (9.3)	168.7 (9.3)	168.8 (9.3)
**Diabetes [b]**						
Yes	20,864 (5.3)	23,228 (5.3)	19,791 (5.2)	18,685 (5.0)	20,788 (5.1)	17,746 (4.9)
**Medications [b]**						
Lipid-lowering	69,732 (17.6)	76,847 (17.6)	66,785 (17.5)	63,141 (17.0)	69,539 (17.0)	60,507 (16.8)
Antiaggregants	61,469 (15.5)	67,698 (15.5)	59,141 (15.5)	55,664 (15.0)	61,234 (15.0)	53,601 (14.9)
**Hypertension [b]**						
No	283,763 (71.6)	312,042 (71.5)	274,493 (71.8)	268,745 (72.2)	295,437 (72.2)	260,129 (72.4)
Untreated	32,496 (8.2)	36,473 (8.4)	30,957 (8.1)	30,500 (8.2)	34,189 (8.4)	29,104 (8.1)
Treated	80,223 (20.2)	87,836 (20.1)	77,047 (20.1)	72,941 (19.6)	79,799 (19.5)	70,097 (19.5)
**Smoking status [b]**						
Never smoked	215,020 (54.2)	236,553 (54.2)	208,727 (54.6)	204,008 (54.8)	224,361 (54.8)	198,112 (55.1)
Former smoker	140,190 (35.4)	153,972 (35.3)	135,254 (35.4)	130,264 (35.0)	143,025 (34.9)	125,761 (35.0)
Current smoker	41,272 (10.4)	45,826 (10.5)	38,516 (10.1)	37,914 (10.2)	42,039 (10.3)	35,457 (9.9)
**Alcohol [b]**						
≤3 times/month	112,252 (28.3)	124,320 (28.5)	107,225 (28.0)	105,238 (28.3)	116,458 (28.4)	100,629 (28.0)
≤4 times/week	200,415 (50.5)	220,095 (50.4)	193,870 (50.7)	188,921 (50.8)	207,416 (50.7)	182,861 (50.9)
Daily	83,815 (21.1)	91,936 (21.1)	81,402 (21.3)	78,027 (21.0)	85,551 (20.9)	75,840 (21.1)
**Physical activity [b]**						
Less active	63,619 (16.0)	70,735 (16.2)	61,315 (16.0)	59,353 (15.9)	65,842 (16.1)	57,266 (15.9)
Moderately active	194,403 (49.0)	213,953 (49.0)	187,205 (48.9)	181,907 (48.9)	200,163 (48.9)	175,332 (48.8)
Very active	138,460 (34.9)	151,663 (34.8)	133,977 (35.0)	130,926 (35.2)	143,420 (35.0)	126,732 (35.3)
**Education [b]**						
Primary	66,930 (16.9)	73,530 (16.9)	63,171 (16.5)	61,126 (16.4)	67,108 (16.4)	57,763 (16.1)
Second/Vocational	201,767 (50.9)	223,449 (51.2)	193,650 (50.6)	190,101 (51.1)	210,420 (51.4)	182,582 (50.8)
University degree	127,785 (32.2)	139,372 (31.9)	125,676 (32.9)	120,959 (32.5)	131,897 (32.2)	118,985 (33.1)
**Dietary intake [c]**						
Fruit | Vegetables	194,159 (49.0)	213,477 (48.9)	187,920 (49.1)	182342 (49.0)	200,426 (49.0)	176,593 (49.1)
Red | Process meat	200,774 (50.6)	220,941 (50.6)	193,232 (50.5)	187499 (50.4)	206,249 (50.4)	180,602 (50.3)
Fibre (>16 g/day)	134,839 (34.0)	147,705 (33.9)	130,907 (34.2)	126653 (34.0)	138,705 (33.9)	123,025 (34.2)
**Biomarkers [d]**						
PLT (*10^9/L)	247 (154−396)	246 (153-396)	247 (154-395)	247 (154−395)	247 (154-395)	247 (154-394)
MPV (fL)	9.3 (7.4−11.6)	9.3 (7.4-11.6)	9.3 (7.4-11.6)	9.3 (7.4−11.6)	9.3 (7.4-11.6)	9.3 (7.4-11.6)
PDW (%)	16.5 (15.5−17.5)	16.5 (15.5-17.5)	16.5 (15.5-17.5)	16.5 (15.5−17.5)	16.5 (15.5-17.5)	16.5 (15.5-17.5)
NEU (*10^9/L)	4.0 (2.1−7.7)	4.0 (2.1-7.7)	4.0 (2.1-7.7)	4.0 (2.1−7.7)	4.0 (2.1-7.7)	4.0 (2.1-7.6)
MO (*10^9/L)	0.45 (0.21−0.94)	0.45 (0.21-0.94)	0.45 (0.21-0.94)	0.44 (0.21−0.93)	0.44 (0.21-0.93)	0.44 (0.21-0.93)
LY (*10^9/L)	1.9 (1.0−3.4)	1.9 (1.0-3.4)	1.8 (1.0-3.4)	1.9 (1.0−3.4)	1.9 (1.0-3.4)	1.8 (1.0-3.4)
CRP (mg/L)	1.4 (0.2−11.0)	1.4 (0.2-11.0)	1.4 (0.2-10.8)	1.4 (0.2−10.7)	1.4 (0.2-10.8)	1.3 (0.2-10.6)

Supplementary Table S2 Characteristics of study participants − legend

ABSI – a body shape index; **BMI** – body mass index; **CRP** – C-reactive protein; **FU** – follow-up time; **HC** – hip circumference; **HI** – hip index; **MPV** – mean platelet volume; **LY** – lymphocyte count; **MO** – monocyte count; **NEU** – neutrophil count; **PDW** – platelet distribution width; **PLT** – platelet count; **n (%)** – number of participants per group (percentage from total per dataset); **n (rate)** – number of kidney cancer cases per group (incidence rate per $1*10^6$ person years); **WC** – waist circumference; **WHI** – waist-to-hip index; **WHR** – waist-to-hip ratio;.

[#] all study participants per dataset – total cases 1086 (263 per $1*10^6$ person years) for the **main** complete-exposures dataset, 1189 (262 per $1*10^6$ person years) for the **extended** dataset, 1042 (262 per $1*10^6$ person years) for the **complete**-covariates dataset.

[a] mean (standard deviation).

[b] number (percent from total per column).

[c] number (percent from total per column) for fruit >3 portions/day or vegetables >5 portions/day; red meat >twice/week or processed meat >once/week; fibre >16 g/day.

[d] geometric mean (95% reference range).

Summaries are shown after sex-specific median imputation for missing covariates.

Covariate missingness for the **main dataset** was as follows: diabetes – 813 (0.2 %); use of lipid-lowering drugs – 2195 (0.6 %); use of antiaggregant/anticoagulants – 3781 (1.0 %); hypertension with or without treatment – 578 (0.1 %); smoking status – 1265 (0.3 % from main dataset); time since quit – 394 (0.3 % from former smokers); cigarettes/day – 1026 (2.5 % from current smokers); alcohol consumption – 285 (<0.1 % from main dataset); physical activity – 1360 (0.3 %); education – 3438 (0.9 %); fruit and vegetable consumption – 375 (<0.1 %); red and processed meat consumption – 116 (<0.1 %); fibre intake – 44 (<0.1 %).

Covariate missingness for the **extended dataset** was as follows: height – 1507 (0.3 % from extended dataset); diabetes – 917 (0.2 %); use of lipid-lowering drugs – 5341 (1.2 %); use of antiaggregant/anticoagulants – 7052 (1.6 %); hypertension & treatment – 671 (0.2 %); smoking status – 1426 (0.3 % from extended dataset); time since quit – 435 (0.3 % from former smokers); cigarettes/day – 1161 (2.5 % from current smokers); alcohol consumption – 315 (<0.1 % from extended dataset); physical activity – 1556 (0.4 %); education – 7090 (1.6 %); fruit and vegetable consumption – 426 (0.1 %); red and processed meat consumption – 133 (<0.1 %); fibre intake – 57 (<0.1 %).

The sex-specific medians used for imputation were category "No" for binary variables (including all individual dietary components); the middle category for three-level variables; 163 cm in women and 176 cm in men for height (extended dataset only); never smoker for women and former smoker for men.

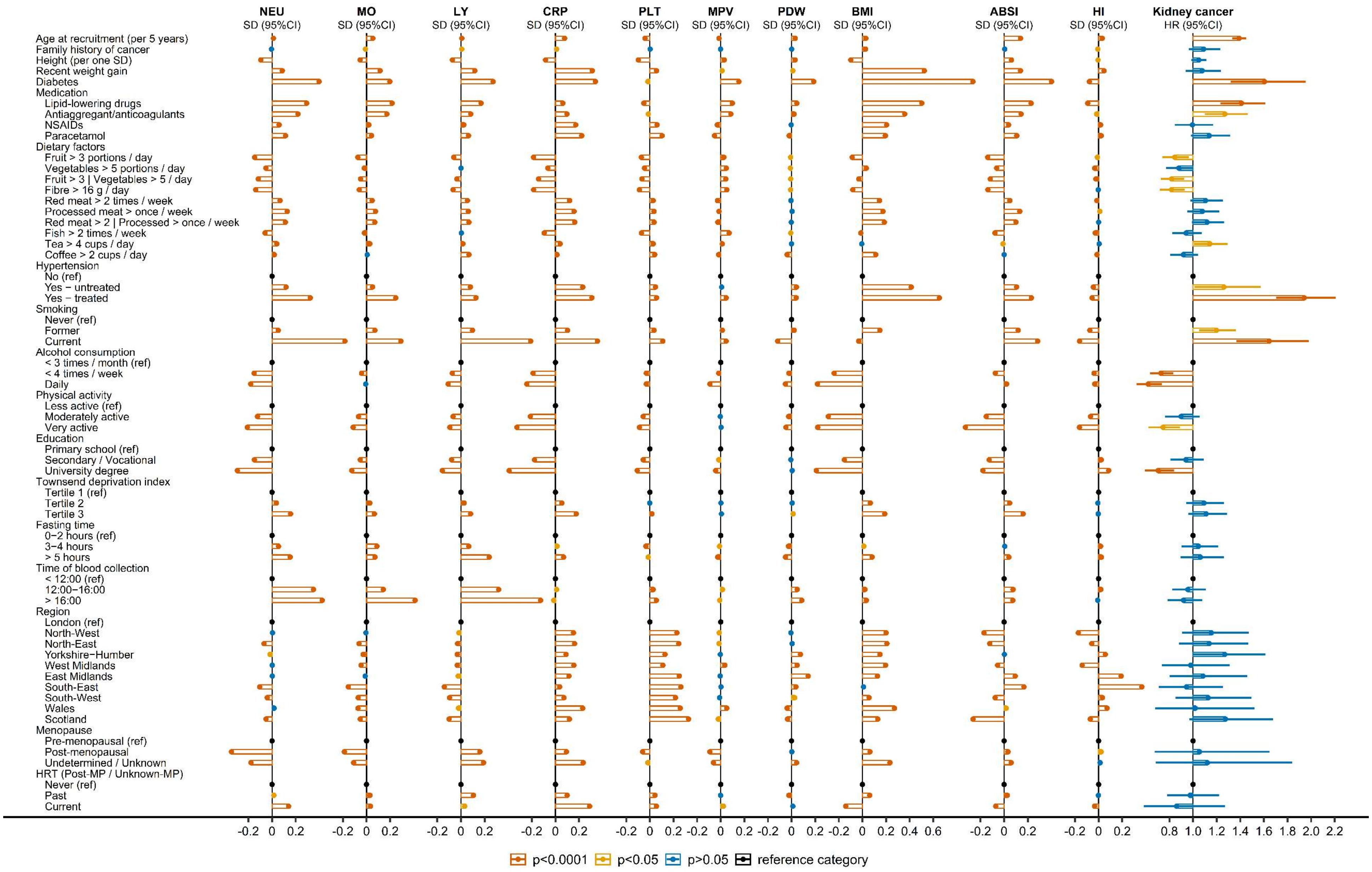

Supplementary Figure S1 Pairwise associations of candidate covariates with the examined exposures and kidney cancer risk

Supplementary Figure S1 Pairwise associations with covariates – legend

ABSI – a body shape index; **BMI** – body mass index; **CRP** – C-reactive protein; **CI** – confidence interval; **HI** – hip index; **HR** – hazard ratio; **HRT** – hormone replacement therapy; **LY** – lymphocyte count; **MO** – monocyte count; **MP** – menopause; **MPV** – mean platelet volume; **NEU** – neutrophil count; **NSAIDs** – non-steroidal anti-inflammatory drugs; **PDW** – platelet distribution width; **PLT** – platelet count; **SD** – standard deviation.

Estimates from liner regression models (SD scale) including individually each exposure specified in the header as the dependent variable and each potential candidate covariate individually as the independent variable, adjusted for age at recruitment (continuous) and sex or from Cox proportional hazards models (HR) with timescale age (or person years of follow-up when examining age at recruitment as exposure) and stratification by sex. Associations with HRT were examined in a subset excluding pre-menopausal women, as very few of them had used HRT.

Covariate categories were defined as described in reference [27], with the following exceptions: recent weight loss and stable weight were combined in one category; family history of bowel, breast, prostate, or lung cancer were combined in one category; **diabetes status** was defined as yes for participants with HbA1c≥48 mmol/mol, as well as for those with self-reported diabetes or use of anti-diabetic medication; **dietary factors** and **education** were defined as described in reference [26], dichotomising dietary factors with respect to the upper cohort-specific tertile boundary.

Family history of cancer (no/yes for bowel, lung, breast, or prostate cancer in parents or siblings), **recent weight gain** within the year prior to recruitment (no/yes, as an indicator of weight dynamics), use of **NSAIDs** or **paracetamol** (no/yes), consumption of **fish** and **coffee**, **Townsend deprivation index** (as a proxy of socio-economic status), **fasting time**, **time of blood collection**, **region** of the assessment centre, and in women **menopausal status** and **HRT use** were omitted from the final analyses because they were not associated with kidney cancer and were thus unlikely to be confounders. **Tea** consumption was also omitted because it had no material associations with the exposures.

The remaining candidate covariates were associated to some extent with the exposures and with kidney cancer risk and were retained in the final analyses. A detailed variable of **smoking status and intensity** was defined as in reference [27] with two categories of never smokers (never smoked (median), just tried), four categories of former smokers (occasional, regular quit ≥20 years (median), regular quit ≥10 years, regular quit <10 years) and three categories for current smoker (occasional, regular ≤10 cigarettes/day (median in women), regular >10 cigarettes/day (median in men)). Missing smoking intensity was imputed with the median for each smoking status category specified above. Combined binary variables were created for **fruit** (>3 portions/day) **or vegetable** (>5 portions/day) **consumption** and **red meat** (>2 times/week) **or processed meat** (>once/week) **consumption** (yes/no if either condition was fulfilled), as each pair showed similar association patterns with the exposures and with kidney cancer risk. **Height** was included as a proxy of lean mass (positively correlated with fat-free mass in UK Biobank [26]), and to account for the residual correlations of the allometric obesity indices with height, because their power coefficients were determined in external datasets. The adjustment for height was retained in the comparisons with traditional body shape indices, to leave only their correlation with BMI as the difference from allometric indices.

A

	cases	Neutrophil count HR (95% CI) (per one SD increase)	p-value	Monocyte count HR (95% CI) (per one SD increase)	p-value
Overall	1141 / 1141	1.31 (1.23 to 1.39)	<0.0001	1.11 (1.05 to 1.18)	0.0005
	1086	1.32 (1.24 to 1.41)	<0.0001	1.12 (1.05 to 1.20)	0.0003
	1086	1.28 (1.20 to 1.37)	<0.0001	1.10 (1.03 to 1.17)	0.003
	1086	1.23 (1.15 to 1.31)	<0.0001	1.06 (1.00 to 1.13)	0.056
	1086	1.21 (1.13 to 1.30)	<0.0001	1.06 (1.00 to 1.13)	0.056
	1141 / 1141	1.20 (1.13 to 1.28)	<0.0001	1.05 (0.99 to 1.12)	0.089
Women	406 / 406	1.42 (1.28 to 1.57)	<0.0001	1.18 (1.06 to 1.31)	0.002
	389	1.42 (1.28 to 1.58)	<0.0001	1.19 (1.07 to 1.32)	0.001
	389	1.39 (1.25 to 1.55)	<0.0001	1.17 (1.05 to 1.30)	0.004
	389	1.32 (1.19 to 1.47)	<0.0001	1.12 (1.01 to 1.25)	0.032
	389	1.29 (1.15 to 1.44)	<0.0001	1.11 (1.00 to 1.24)	0.045
	406 / 406	1.28 (1.15 to 1.43)	<0.0001	1.11 (1.00 to 1.23)	0.051
Men	735 / 735	1.25 (1.16 to 1.35)	<0.0001	1.08 (1.00 to 1.17)	0.041
	697	1.27 (1.17 to 1.37)	<0.0001	1.09 (1.01 to 1.18)	0.029
	697	1.22 (1.13 to 1.33)	<0.0001	1.07 (0.99 to 1.15)	0.108
	697	1.18 (1.09 to 1.28)	<0.0001	1.03 (0.96 to 1.11)	0.426
	697	1.17 (1.08 to 1.27)	0.0002	1.03 (0.96 to 1.12)	0.385
	735 / 735	1.16 (1.07 to 1.25)	0.0004	1.03 (0.95 to 1.10)	0.511
FU<6 years	527 / 527	1.31 (1.19 to 1.43)	<0.0001	1.09 (1.00 to 1.19)	0.060
	504	1.31 (1.20 to 1.44)	<0.0001	1.09 (1.00 to 1.19)	0.062
	504	1.29 (1.17 to 1.42)	<0.0001	1.07 (0.98 to 1.18)	0.130
	504	1.23 (1.12 to 1.35)	<0.0001	1.03 (0.94 to 1.13)	0.496
	504	1.22 (1.11 to 1.34)	<0.0001	1.03 (0.94 to 1.13)	0.473
	527 / 527	1.22 (1.11 to 1.34)	<0.0001	1.03 (0.95 to 1.13)	0.475
FU≥6 years	614 / 614	1.32 (1.21 to 1.44)	<0.0001	1.14 (1.05 to 1.24)	0.002
	582	1.34 (1.22 to 1.46)	<0.0001	1.16 (1.06 to 1.26)	0.0008
	582	1.28 (1.17 to 1.40)	<0.0001	1.13 (1.04 to 1.23)	0.005
	582	1.23 (1.13 to 1.35)	<0.0001	1.10 (1.01 to 1.20)	0.035
	582	1.21 (1.11 to 1.33)	<0.0001	1.09 (1.00 to 1.19)	0.052
	614 / 614	1.19 (1.09 to 1.30)	<0.0001	1.07 (0.99 to 1.17)	0.097

	cases	C-reactive protein HR (95% CI)	p-value	Lymphocyte count HR (95% CI)	p-value
Overall	1113 / 1141	1.33 (1.26 to 1.41)	<0.0001	1.02 (0.96 to 1.08)	0.473
	1086	1.33 (1.26 to 1.41)	<0.0001	1.03 (0.97 to 1.09)	0.342
	1086	1.31 (1.23 to 1.39)	<0.0001	0.99 (0.93 to 1.05)	0.770
	1086	1.27 (1.20 to 1.35)	<0.0001	0.98 (0.92 to 1.04)	0.438
	1086	1.25 (1.18 to 1.33)	<0.0001	0.97 (0.91 to 1.03)	0.291
	1113 / 1141	1.25 (1.18 to 1.33)	<0.0001	0.96 (0.91 to 1.02)	0.179
Women	397 / 406	1.39 (1.26 to 1.53)	<0.0001	1.10 (1.00 to 1.22)	0.055
	389	1.40 (1.27 to 1.54)	<0.0001	1.11 (1.00 to 1.22)	0.046
	389	1.38 (1.25 to 1.52)	<0.0001	1.07 (0.97 to 1.19)	0.191
	389	1.31 (1.19 to 1.45)	<0.0001	1.05 (0.94 to 1.16)	0.394
	389	1.29 (1.16 to 1.43)	<0.0001	1.03 (0.93 to 1.14)	0.627
	397 / 406	1.28 (1.16 to 1.42)	<0.0001	1.02 (0.92 to 1.13)	0.703
Men	716 / 735	1.31 (1.22 to 1.40)	<0.0001	0.98 (0.91 to 1.05)	0.593
	697	1.30 (1.21 to 1.40)	<0.0001	0.99 (0.92 to 1.07)	0.763
	697	1.27 (1.18 to 1.37)	<0.0001	0.95 (0.88 to 1.02)	0.177
	697	1.25 (1.16 to 1.34)	<0.0001	0.94 (0.87 to 1.01)	0.101
	697	1.23 (1.14 to 1.32)	<0.0001	0.93 (0.87 to 1.01)	0.078
	716 / 735	1.23 (1.14 to 1.32)	<0.0001	0.93 (0.86 to 1.00)	0.040
FU<6 years	518 / 527	1.46 (1.34 to 1.58)	<0.0001	0.96 (0.89 to 1.05)	0.399
	504	1.47 (1.35 to 1.59)	<0.0001	0.98 (0.89 to 1.06)	0.572
	504	1.45 (1.33 to 1.58)	<0.0001	0.95 (0.87 to 1.03)	0.228
	504	1.41 (1.29 to 1.53)	<0.0001	0.93 (0.86 to 1.02)	0.118
	504	1.39 (1.27 to 1.51)	<0.0001	0.93 (0.85 to 1.01)	0.092
	518 / 527	1.38 (1.27 to 1.50)	<0.0001	0.92 (0.85 to 1.00)	0.050
FU≥6 years	595 / 614	1.24 (1.14 to 1.34)	<0.0001	1.08 (0.99 to 1.17)	0.078
	582	1.23 (1.13 to 1.33)	<0.0001	1.08 (0.99 to 1.17)	0.067
	582	1.20 (1.10 to 1.30)	<0.0001	1.03 (0.95 to 1.12)	0.470
	582	1.16 (1.07 to 1.26)	0.0003	1.02 (0.93 to 1.11)	0.700
	582	1.14 (1.05 to 1.24)	0.002	1.00 (0.92 to 1.09)	0.910
	595 / 614	1.15 (1.05 to 1.24)	0.001	1.00 (0.92 to 1.08)	0.975

0.8 1.0 1.2 1.4 1.6 — 0.8 1.0 1.2 1.4

Sex (extended) ▲ Sex (main) ✳ + Smoking ■ + Hypertension ● Fully adjusted (main) ○ Fully adjusted (extended)

Supplementary Figure S2A Sequential adjustment for covariates − associations of leucocyte counts and CRP with kidney cancer risk

B

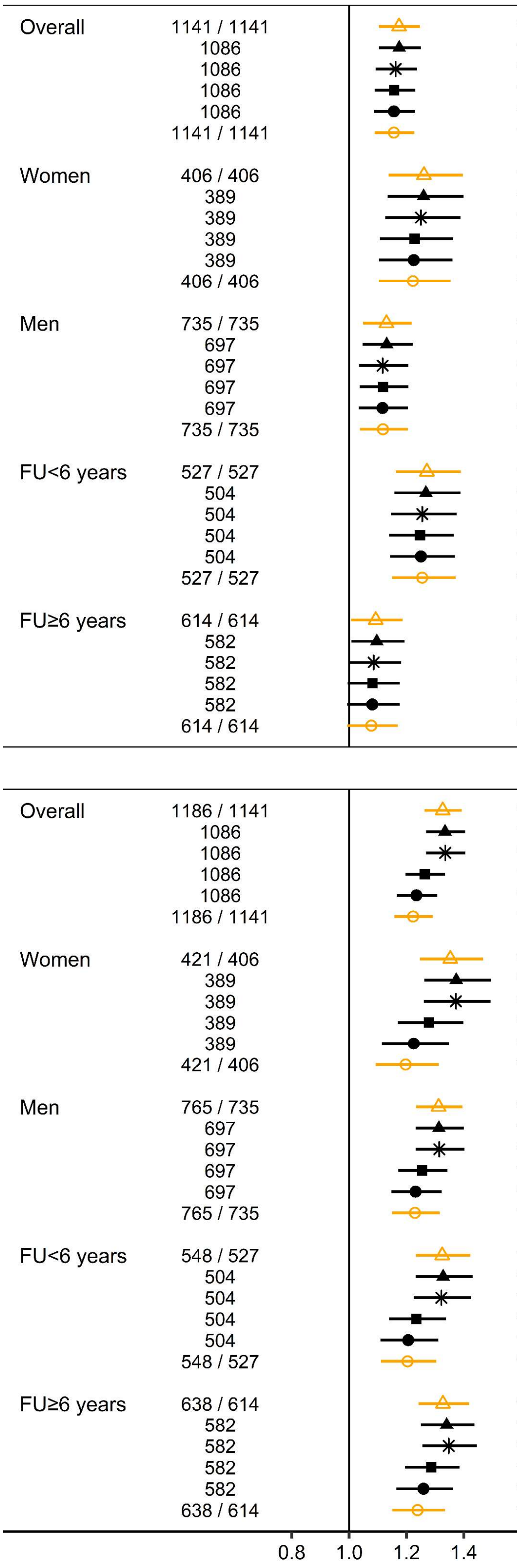
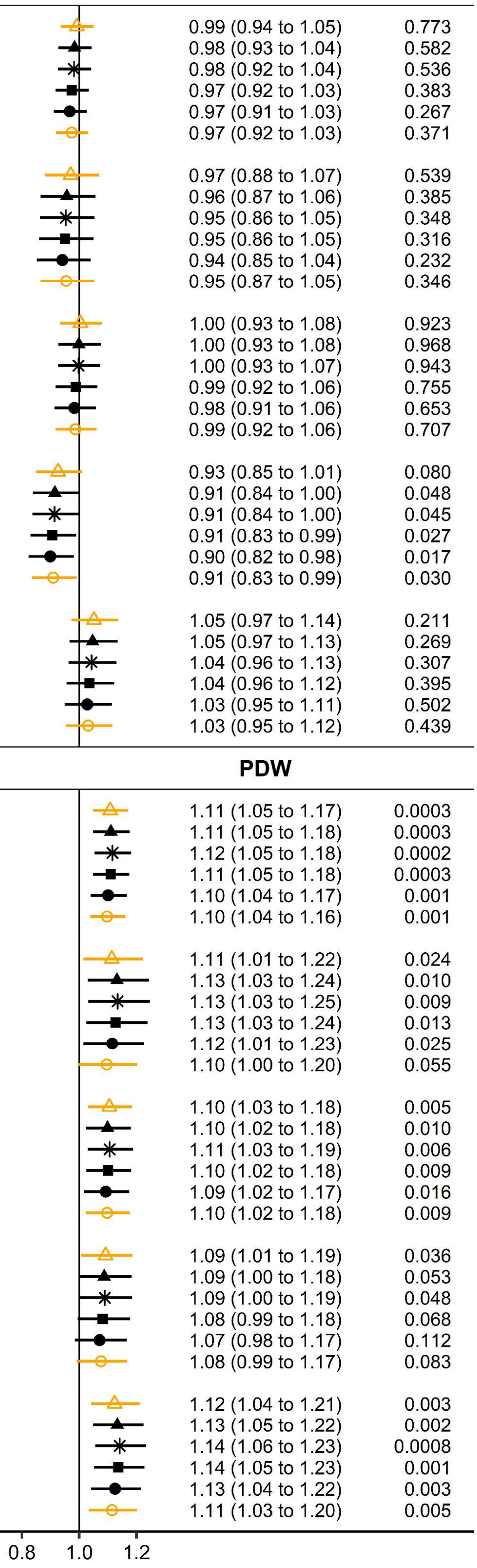

	cases	Platelet count HR (95% CI) (per one SD increase)	Platelet count p-value	MPV HR (95% CI) (per one SD increase)	MPV p-value
Overall	1141 / 1141	1.17 (1.11 to 1.25)	<0.0001	0.99 (0.94 to 1.05)	0.773
	1086	1.17 (1.10 to 1.25)	<0.0001	0.98 (0.93 to 1.04)	0.582
	1086	1.16 (1.09 to 1.24)	<0.0001	0.98 (0.92 to 1.04)	0.536
	1086	1.16 (1.09 to 1.23)	<0.0001	0.97 (0.92 to 1.03)	0.383
	1086	1.16 (1.09 to 1.23)	<0.0001	0.97 (0.91 to 1.03)	0.267
	1141 / 1141	1.16 (1.09 to 1.23)	<0.0001	0.97 (0.92 to 1.03)	0.371
Women	406 / 406	1.26 (1.14 to 1.40)	<0.0001	0.97 (0.88 to 1.07)	0.539
	389	1.26 (1.13 to 1.40)	<0.0001	0.96 (0.87 to 1.06)	0.385
	389	1.25 (1.13 to 1.39)	<0.0001	0.95 (0.86 to 1.05)	0.348
	389	1.23 (1.11 to 1.36)	0.0001	0.95 (0.86 to 1.05)	0.316
	389	1.23 (1.10 to 1.36)	0.0001	0.94 (0.85 to 1.04)	0.232
	406 / 406	1.22 (1.10 to 1.35)	0.0001	0.95 (0.87 to 1.05)	0.346
Men	735 / 735	1.13 (1.05 to 1.22)	0.001	1.00 (0.93 to 1.08)	0.923
	697	1.13 (1.05 to 1.22)	0.002	1.00 (0.93 to 1.08)	0.968
	697	1.12 (1.03 to 1.21)	0.005	1.00 (0.93 to 1.07)	0.943
	697	1.12 (1.04 to 1.21)	0.004	0.99 (0.92 to 1.06)	0.755
	697	1.12 (1.03 to 1.21)	0.005	0.98 (0.91 to 1.06)	0.653
	735 / 735	1.12 (1.04 to 1.20)	0.003	0.99 (0.92 to 1.06)	0.707
FU<6 years	527 / 527	1.27 (1.16 to 1.39)	<0.0001	0.93 (0.85 to 1.01)	0.080
	504	1.27 (1.16 to 1.39)	<0.0001	0.91 (0.84 to 1.00)	0.048
	504	1.26 (1.15 to 1.37)	<0.0001	0.91 (0.84 to 1.00)	0.045
	504	1.25 (1.14 to 1.37)	<0.0001	0.91 (0.83 to 0.99)	0.027
	504	1.25 (1.14 to 1.37)	<0.0001	0.90 (0.82 to 0.98)	0.017
	527 / 527	1.26 (1.15 to 1.37)	<0.0001	0.91 (0.83 to 0.99)	0.030
FU≥6 years	614 / 614	1.09 (1.01 to 1.19)	0.034	1.05 (0.97 to 1.14)	0.211
	582	1.10 (1.01 to 1.19)	0.033	1.05 (0.97 to 1.13)	0.269
	582	1.09 (1.00 to 1.18)	0.056	1.04 (0.96 to 1.13)	0.307
	582	1.08 (0.99 to 1.18)	0.066	1.04 (0.96 to 1.12)	0.395
	582	1.08 (0.99 to 1.18)	0.070	1.03 (0.95 to 1.11)	0.502
	614 / 614	1.08 (0.99 to 1.17)	0.072	1.03 (0.95 to 1.12)	0.439

	cases	BMI HR (95% CI)	BMI p-value	PDW HR (95% CI)	PDW p-value
Overall	1186 / 1141	1.33 (1.26 to 1.39)	<0.0001	1.11 (1.05 to 1.17)	0.0003
	1086	1.33 (1.27 to 1.40)	<0.0001	1.11 (1.05 to 1.18)	0.0003
	1086	1.34 (1.27 to 1.41)	<0.0001	1.12 (1.05 to 1.18)	0.0002
	1086	1.26 (1.20 to 1.33)	<0.0001	1.11 (1.05 to 1.18)	0.0003
	1086	1.23 (1.17 to 1.31)	<0.0001	1.10 (1.04 to 1.17)	0.001
	1186 / 1141	1.22 (1.16 to 1.29)	<0.0001	1.10 (1.04 to 1.16)	0.001
Women	421 / 406	1.35 (1.25 to 1.47)	<0.0001	1.11 (1.01 to 1.22)	0.024
	389	1.37 (1.26 to 1.50)	<0.0001	1.13 (1.03 to 1.24)	0.010
	389	1.37 (1.26 to 1.49)	<0.0001	1.13 (1.03 to 1.25)	0.009
	389	1.28 (1.17 to 1.40)	<0.0001	1.13 (1.03 to 1.24)	0.013
	389	1.23 (1.11 to 1.35)	<0.0001	1.12 (1.01 to 1.23)	0.025
	421 / 406	1.20 (1.09 to 1.31)	0.0001	1.10 (1.00 to 1.20)	0.055
Men	765 / 735	1.31 (1.23 to 1.39)	<0.0001	1.10 (1.03 to 1.18)	0.005
	697	1.31 (1.23 to 1.40)	<0.0001	1.10 (1.02 to 1.18)	0.010
	697	1.31 (1.23 to 1.40)	<0.0001	1.11 (1.03 to 1.19)	0.006
	697	1.25 (1.17 to 1.34)	<0.0001	1.10 (1.02 to 1.18)	0.009
	697	1.23 (1.15 to 1.32)	<0.0001	1.09 (1.02 to 1.17)	0.016
	765 / 735	1.23 (1.15 to 1.32)	<0.0001	1.10 (1.02 to 1.18)	0.009
FU<6 years	548 / 527	1.32 (1.23 to 1.42)	<0.0001	1.09 (1.01 to 1.19)	0.036
	504	1.33 (1.23 to 1.43)	<0.0001	1.09 (1.00 to 1.18)	0.053
	504	1.32 (1.23 to 1.43)	<0.0001	1.09 (1.00 to 1.19)	0.048
	504	1.24 (1.14 to 1.34)	<0.0001	1.08 (0.99 to 1.18)	0.068
	504	1.21 (1.11 to 1.31)	<0.0001	1.07 (0.98 to 1.17)	0.112
	548 / 527	1.20 (1.11 to 1.30)	<0.0001	1.08 (0.99 to 1.17)	0.083
FU≥6 years	638 / 614	1.33 (1.24 to 1.42)	<0.0001	1.12 (1.04 to 1.21)	0.003
	582	1.34 (1.25 to 1.44)	<0.0001	1.13 (1.05 to 1.22)	0.002
	582	1.35 (1.26 to 1.45)	<0.0001	1.14 (1.06 to 1.23)	0.0008
	582	1.29 (1.20 to 1.38)	<0.0001	1.14 (1.05 to 1.23)	0.001
	582	1.26 (1.17 to 1.36)	<0.0001	1.13 (1.04 to 1.22)	0.003
	638 / 614	1.24 (1.15 to 1.33)	<0.0001	1.11 (1.03 to 1.20)	0.005

Sex (extended) · Sex (main) · + Smoking · + Hypertension · Fully adjusted (main) · Fully adjusted (extended)

Supplementary Figure S2B Sequential adjustment for covariates − associations of platelet parameters and BMI with kidney cancer risk

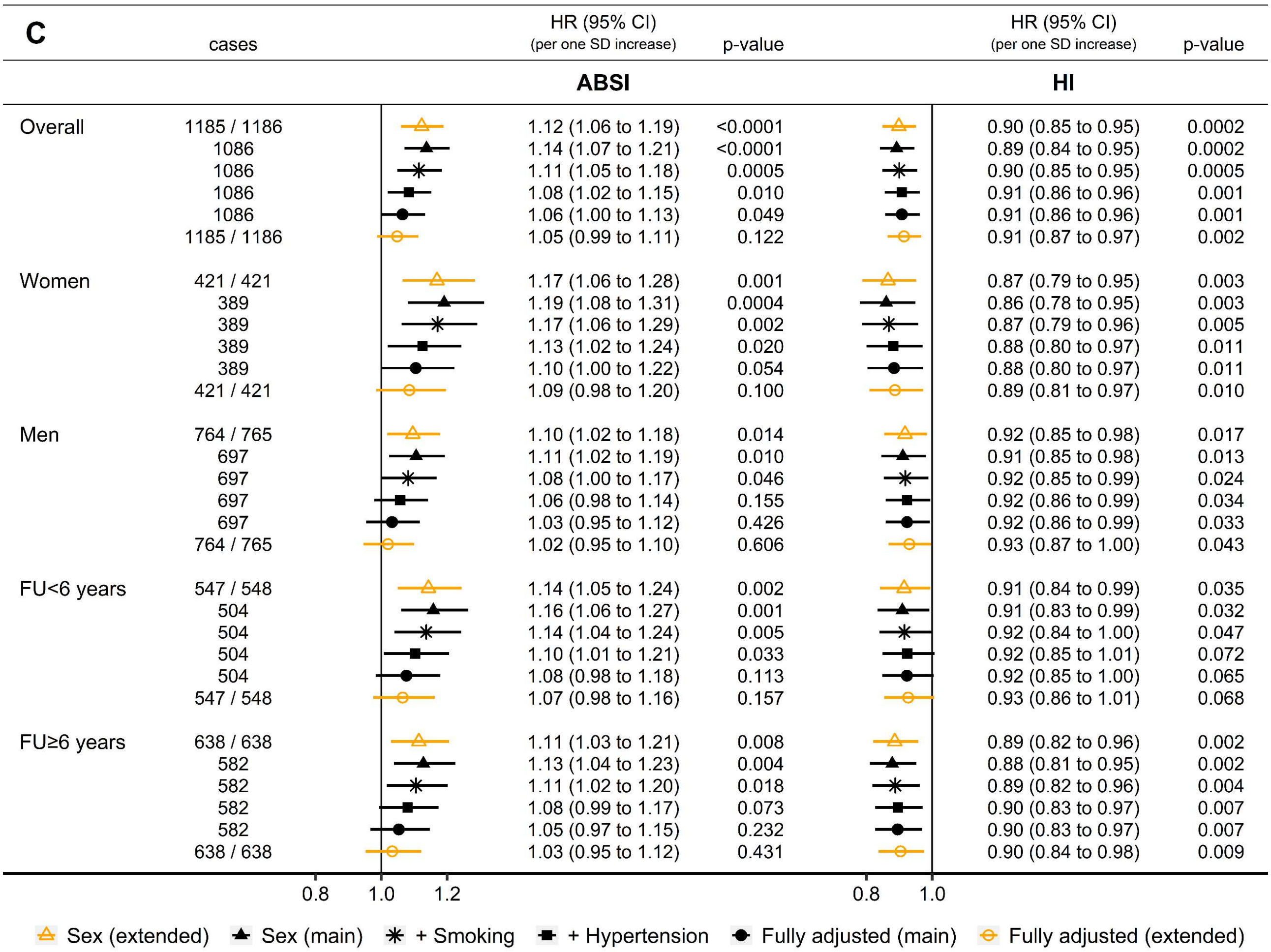

C	cases	ABSI HR (95% CI) (per one SD increase)	ABSI p-value	HI HR (95% CI) (per one SD increase)	HI p-value
Overall	1185 / 1186	1.12 (1.06 to 1.19)	<0.0001	0.90 (0.85 to 0.95)	0.0002
	1086	1.14 (1.07 to 1.21)	<0.0001	0.89 (0.84 to 0.95)	0.0002
	1086	1.11 (1.05 to 1.18)	0.0005	0.90 (0.85 to 0.95)	0.0005
	1086	1.08 (1.02 to 1.15)	0.010	0.91 (0.86 to 0.96)	0.001
	1086	1.06 (1.00 to 1.13)	0.049	0.91 (0.86 to 0.96)	0.001
	1185 / 1186	1.05 (0.99 to 1.11)	0.122	0.91 (0.87 to 0.97)	0.002
Women	421 / 421	1.17 (1.06 to 1.28)	0.001	0.87 (0.79 to 0.95)	0.003
	389	1.19 (1.08 to 1.31)	0.0004	0.86 (0.78 to 0.95)	0.003
	389	1.17 (1.06 to 1.29)	0.002	0.87 (0.79 to 0.96)	0.005
	389	1.13 (1.02 to 1.24)	0.020	0.88 (0.80 to 0.97)	0.011
	389	1.10 (1.00 to 1.22)	0.054	0.88 (0.80 to 0.97)	0.011
	421 / 421	1.09 (0.98 to 1.20)	0.100	0.89 (0.81 to 0.97)	0.010
Men	764 / 765	1.10 (1.02 to 1.18)	0.014	0.92 (0.85 to 0.98)	0.017
	697	1.11 (1.02 to 1.19)	0.010	0.91 (0.85 to 0.98)	0.013
	697	1.08 (1.00 to 1.17)	0.046	0.92 (0.85 to 0.99)	0.024
	697	1.06 (0.98 to 1.14)	0.155	0.92 (0.86 to 0.99)	0.034
	697	1.03 (0.95 to 1.12)	0.426	0.92 (0.86 to 0.99)	0.033
	764 / 765	1.02 (0.95 to 1.10)	0.606	0.93 (0.87 to 1.00)	0.043
FU<6 years	547 / 548	1.14 (1.05 to 1.24)	0.002	0.91 (0.84 to 0.99)	0.035
	504	1.16 (1.06 to 1.27)	0.001	0.91 (0.83 to 0.99)	0.032
	504	1.14 (1.04 to 1.24)	0.005	0.92 (0.84 to 1.00)	0.047
	504	1.10 (1.01 to 1.21)	0.033	0.92 (0.85 to 1.01)	0.072
	504	1.08 (0.98 to 1.18)	0.113	0.92 (0.85 to 1.00)	0.065
	547 / 548	1.07 (0.98 to 1.16)	0.157	0.93 (0.86 to 1.01)	0.068
FU≥6 years	638 / 638	1.11 (1.03 to 1.21)	0.008	0.89 (0.82 to 0.96)	0.002
	582	1.13 (1.04 to 1.23)	0.004	0.88 (0.81 to 0.95)	0.002
	582	1.11 (1.02 to 1.20)	0.018	0.89 (0.82 to 0.96)	0.004
	582	1.08 (0.99 to 1.17)	0.073	0.90 (0.83 to 0.97)	0.007
	582	1.05 (0.97 to 1.15)	0.232	0.90 (0.83 to 0.97)	0.007
	638 / 638	1.03 (0.95 to 1.12)	0.431	0.90 (0.84 to 0.98)	0.009

Supplementary Figure S2C Sequential adjustment for covariates – associations of allometric body shape indices with kidney cancer risk

ABSI – a body shape index; **BMI** – body mass index; **CI** – confidence interval; **CRP** – C-reactive protein; **HI** – hip index; **HR** – hazard ratio; **MPV** – mean platelet volume; **PDW** – platelet distribution width; **SD** – standard deviation; **cases** – number of kidney cancer cases (left / right column); **p-value** – Wald test for the individual term; **FU<6 years** – cases diagnosed within the first 6 years of follow-up, censoring follow-up at 6 years; **FU≥6 years** – cases diagnosed after 6 years of follow-up, lagging entry time with 6 years and excluding participants with follow-up<6 years.

Cox proportional hazards models examining each exposure individually (sex-specific z-scores, value minus mean divided by standard deviation, after log-transformation for biomarkers) with sequential introduction of stratifications and adjustments as follows:

Sex (extended) – stratified by sex (except for sex-specific models) in the extended dataset, omitting exclusions for missing exposures and using all cohort participants with available measurements of the corresponding exposure.

Sex (main) – stratified by sex (except for sex-specific models) in the main complete-exposures dataset.

Supplementary Figure S2 Sequential adjustment for covariates – legend (continued)

+ Smoking – further adding stratification for smoking status and intensity to the model stratified by sex in the main dataset.

+ Hypertension – further adjusting for hypertension (with or without treatment) of the model stratified by sex and smoking status and intensity in the main dataset.

Fully adjusted (main) – the main multivariable model for individual exposures, stratified by age at recruitment, smoking status and intensity, and sex, and adjusted for height (sex-specific z-scores), alcohol consumption, physical activity, education, diabetes, use of lipid-lowering drugs, hypertension (with or without treatment), use of antiaggregant/anticoagulants, and dietary intake of fruit and vegetables, red and processed meat, and fibre (shown in Figure 2 for leucocyte counts and CRP, Figure 3 for platelet parameters, and Figure 4 for allometric obesity indices).

Fully adjusted (extended) – a fully adjusted model (as above) for individual exposures in the extended dataset, omitting exclusions for missing exposures and using all cohort participants with available measurements of the corresponding exposure.

Number of cohort participants in each model:

	Overall	Women	Men	FU<6 years	FU≥6 years
Main dataset	396,482	211,843	184,639	396,482	372,186
Extended dataset					
Leucocyte counts	415,904	222,101	193,803	415,904	390,330
Platelet count	416,630	222,491	194,139	416,630	391,011
MPV & PDW	416,625	222,488	194,137	416,625	391,006
CRP	408,568	218,587	189,981	408,568	383,417
BMI	434,397	232,989	201,408	434,397	407,691
ABSI	434,337	232,954	201,383	434,337	407,637
HI	434,342	232,958	201,384	434,342	407,641

A

	Overall HR (95% CI) (per one SD increase)	Women HR (95% CI) (per one SD increase)	Men HR (95% CI) (per one SD increase)	FU < 6 years HR (95% CI) (per one SD increase)	FU ≥ 6 years HR (95% CI) (per one SD increase)
**Neutrophil count**					
Individual exposure	1.21 (1.13 to 1.30)	1.29 (1.15 to 1.44)	1.17 (1.08 to 1.27)	1.22 (1.11 to 1.34)	1.21 (1.11 to 1.33)
All WBC	1.21 (1.13 to 1.30)	1.27 (1.14 to 1.43)	1.18 (1.08 to 1.28)	1.23 (1.11 to 1.36)	1.20 (1.09 to 1.32)
All WBC + CRP	1.15 (1.08 to 1.24)	1.20 (1.07 to 1.35)	1.13 (1.03 to 1.23)	1.13 (1.03 to 1.25)	1.17 (1.07 to 1.29)
All WBC + CRP + PLT	1.13 (1.05 to 1.21)	1.17 (1.04 to 1.32)	1.11 (1.02 to 1.21)	1.09 (0.99 to 1.21)	1.17 (1.06 to 1.28)
All WBC + CRP + PLT parameters	1.12 (1.05 to 1.20)	1.16 (1.03 to 1.31)	1.10 (1.01 to 1.20)	1.09 (0.99 to 1.21)	1.15 (1.05 to 1.27)
All exposures	1.12 (1.04 to 1.20)	1.16 (1.03 to 1.31)	1.10 (1.01 to 1.20)	1.09 (0.98 to 1.20)	1.15 (1.05 to 1.27)
**Monocyte count**					
Individual exposure	1.06 (1.00 to 1.13)	1.11 (1.00 to 1.24)	1.03 (0.96 to 1.12)	1.03 (0.94 to 1.13)	1.09 (1.00 to 1.19)
+ NEU	1.02 (0.96 to 1.09)	1.06 (0.95 to 1.18)	1.00 (0.93 to 1.08)	0.99 (0.91 to 1.09)	1.05 (0.96 to 1.14)
All WBC	1.04 (0.98 to 1.11)	1.07 (0.96 to 1.20)	1.03 (0.95 to 1.12)	1.03 (0.93 to 1.13)	1.06 (0.97 to 1.16)
All WBC + CRP	1.02 (0.96 to 1.09)	1.06 (0.95 to 1.18)	1.01 (0.93 to 1.09)	0.99 (0.91 to 1.09)	1.05 (0.96 to 1.15)
All WBC + CRP + PLT parameters	1.03 (0.96 to 1.09)	1.06 (0.95 to 1.18)	1.01 (0.93 to 1.10)	1.00 (0.91 to 1.09)	1.05 (0.96 to 1.15)
All exposures	1.02 (0.96 to 1.09)	1.06 (0.95 to 1.18)	1.00 (0.92 to 1.09)	0.99 (0.91 to 1.09)	1.04 (0.95 to 1.14)
**Lymphocyte count**					
Individual exposure	0.97 (0.91 to 1.03)	1.03 (0.93 to 1.14)	0.93 (0.87 to 1.01)	0.93 (0.85 to 1.01)	1.00 (0.92 to 1.09)
+ NEU	0.94 (0.89 to 1.00)	0.98 (0.89 to 1.09)	0.92 (0.85 to 0.99)	0.90 (0.83 to 0.99)	0.98 (0.90 to 1.06)
All WBC	0.93 (0.87 to 0.99)	0.96 (0.87 to 1.07)	0.91 (0.84 to 0.98)	0.90 (0.82 to 0.98)	0.96 (0.88 to 1.05)
All WBC + CRP	0.94 (0.88 to 1.00)	0.96 (0.87 to 1.07)	0.92 (0.85 to 0.99)	0.91 (0.83 to 1.00)	0.96 (0.88 to 1.05)
All WBC + CRP + PLT parameters	0.93 (0.87 to 0.99)	0.95 (0.86 to 1.06)	0.91 (0.84 to 0.99)	0.90 (0.82 to 0.98)	0.96 (0.88 to 1.05)
All WBC + CRP + PLT param + BMI	0.91 (0.85 to 0.96)	0.94 (0.84 to 1.04)	0.88 (0.82 to 0.96)	0.88 (0.80 to 0.96)	0.93 (0.85 to 1.02)
All exposures	0.90 (0.84 to 0.96)	0.93 (0.83 to 1.03)	0.88 (0.81 to 0.95)	0.88 (0.80 to 0.96)	0.92 (0.85 to 1.01)
**C-reactive protein**					
Individual exposure	1.25 (1.18 to 1.33)	1.29 (1.16 to 1.43)	1.23 (1.14 to 1.32)	1.39 (1.27 to 1.51)	1.14 (1.05 to 1.24)
+ NEU	1.21 (1.14 to 1.29)	1.22 (1.10 to 1.36)	1.20 (1.11 to 1.29)	1.35 (1.23 to 1.48)	1.10 (1.01 to 1.20)
+ all WBC	1.21 (1.13 to 1.28)	1.22 (1.09 to 1.35)	1.19 (1.10 to 1.29)	1.34 (1.23 to 1.47)	1.09 (1.00 to 1.19)
+ all WBC + PLT	1.20 (1.12 to 1.27)	1.20 (1.08 to 1.34)	1.19 (1.10 to 1.28)	1.33 (1.21 to 1.45)	1.09 (1.00 to 1.19)
+ all WBC + PLT parameters	1.19 (1.11 to 1.26)	1.19 (1.07 to 1.32)	1.18 (1.09 to 1.27)	1.31 (1.20 to 1.44)	1.08 (0.99 to 1.18)
+ all WBC + PLT parameters + BMI	1.12 (1.05 to 1.20)	1.12 (0.99 to 1.26)	1.12 (1.03 to 1.22)	1.26 (1.15 to 1.39)	1.00 (0.91 to 1.10)
All exposures	1.11 (1.04 to 1.19)	1.11 (0.98 to 1.25)	1.12 (1.03 to 1.21)	1.26 (1.14 to 1.38)	0.99 (0.90 to 1.09)

Axis: 0.8 1.0 1.2 1.4 (Overall, Women, Men, FU ≥ 6 years); 0.8 1.0 1.2 1.4 1.6 (FU < 6 years)

Legend: Individual exposures; Adding NEU; Adding all WBC; Adding CRP; Adding PLT; Adding all PLT parameters; Adding BMI; All exposures combined

Supplementary Figure S3A Sequential addition of exposures – associations of leucocyte counts and CRP with kidney cancer risk

B	Overall HR (95% CI) (per one SD increase)	Women HR (95% CI) (per one SD increase)	Men HR (95% CI) (per one SD increase)	FU < 6 years HR (95% CI) (per one SD increase)	FU ≥ 6 years HR (95% CI) (per one SD increase)
**Platelet count**					
Individual exposure	1.16 (1.09 to 1.23)	1.23 (1.10 to 1.36)	1.12 (1.03 to 1.21)	1.25 (1.14 to 1.37)	1.08 (0.99 to 1.18)
+ PDW	1.23 (1.15 to 1.32)	1.32 (1.18 to 1.47)	1.18 (1.09 to 1.28)	1.33 (1.21 to 1.46)	1.16 (1.06 to 1.26)
All PLT parameters	1.23 (1.14 to 1.32)	1.31 (1.16 to 1.48)	1.18 (1.08 to 1.29)	1.30 (1.17 to 1.44)	1.17 (1.06 to 1.29)
All PLT parameters + NEU	1.17 (1.09 to 1.26)	1.23 (1.09 to 1.39)	1.14 (1.04 to 1.25)	1.24 (1.12 to 1.38)	1.12 (1.01 to 1.23)
All PLT parameters + WBC	1.18 (1.10 to 1.27)	1.23 (1.09 to 1.40)	1.15 (1.05 to 1.26)	1.26 (1.13 to 1.40)	1.12 (1.01 to 1.23)
All PLT parameters + WBC + CRP	1.16 (1.08 to 1.25)	1.21 (1.07 to 1.37)	1.14 (1.04 to 1.25)	1.23 (1.10 to 1.37)	1.11 (1.00 to 1.23)
All exposures combined	1.18 (1.10 to 1.27)	1.21 (1.07 to 1.38)	1.16 (1.06 to 1.28)	1.24 (1.12 to 1.38)	1.13 (1.02 to 1.25)
**MPV**					
Individual exposure	0.97 (0.91 to 1.03)	0.94 (0.85 to 1.04)	0.98 (0.91 to 1.06)	0.90 (0.82 to 0.98)	1.03 (0.95 to 1.11)
+ PLT	1.05 (0.98 to 1.12)	1.05 (0.94 to 1.18)	1.05 (0.96 to 1.14)	1.00 (0.90 to 1.10)	1.09 (0.99 to 1.19)
All PLT parameters	0.99 (0.92 to 1.06)	0.98 (0.87 to 1.10)	1.00 (0.91 to 1.09)	0.94 (0.85 to 1.04)	1.03 (0.94 to 1.14)
All PLT parameters + WBC	0.97 (0.91 to 1.04)	0.95 (0.84 to 1.07)	0.99 (0.90 to 1.08)	0.93 (0.84 to 1.03)	1.01 (0.92 to 1.11)
All PLT parameters + WBC + CRP	0.98 (0.91 to 1.05)	0.95 (0.84 to 1.07)	0.99 (0.91 to 1.08)	0.94 (0.85 to 1.04)	1.01 (0.92 to 1.11)
All exposures combined	0.98 (0.91 to 1.05)	0.96 (0.85 to 1.08)	1.00 (0.91 to 1.09)	0.94 (0.85 to 1.05)	1.01 (0.92 to 1.12)
**PDW**					
Individual exposure	1.10 (1.04 to 1.17)	1.12 (1.01 to 1.23)	1.09 (1.02 to 1.17)	1.07 (0.98 to 1.17)	1.13 (1.04 to 1.22)
+ PLT	1.19 (1.12 to 1.26)	1.23 (1.11 to 1.36)	1.16 (1.07 to 1.25)	1.18 (1.08 to 1.29)	1.19 (1.09 to 1.29)
All PLT parameters	1.19 (1.12 to 1.27)	1.24 (1.11 to 1.38)	1.16 (1.07 to 1.26)	1.20 (1.09 to 1.32)	1.18 (1.08 to 1.28)
All PLT parameters + WBC	1.18 (1.11 to 1.26)	1.23 (1.10 to 1.37)	1.16 (1.07 to 1.25)	1.19 (1.09 to 1.31)	1.17 (1.07 to 1.28)
All PLT parameters + WBC + CRP	1.17 (1.10 to 1.25)	1.22 (1.09 to 1.36)	1.15 (1.06 to 1.24)	1.17 (1.07 to 1.29)	1.17 (1.07 to 1.27)
All exposures combined	1.16 (1.09 to 1.24)	1.21 (1.08 to 1.34)	1.14 (1.05 to 1.23)	1.17 (1.06 to 1.28)	1.16 (1.06 to 1.26)
**BMI**					
Individual exposure	1.23 (1.17 to 1.31)	1.23 (1.11 to 1.35)	1.23 (1.15 to 1.32)	1.21 (1.11 to 1.31)	1.26 (1.17 to 1.36)
+ WBC	1.23 (1.16 to 1.30)	1.20 (1.09 to 1.32)	1.24 (1.15 to 1.33)	1.21 (1.11 to 1.31)	1.25 (1.15 to 1.35)
+ WBC + CRP	1.18 (1.11 to 1.25)	1.14 (1.02 to 1.26)	1.20 (1.11 to 1.29)	1.12 (1.02 to 1.22)	1.24 (1.14 to 1.35)
+ WBC + CRP + PLT parameters	1.19 (1.12 to 1.26)	1.14 (1.03 to 1.27)	1.21 (1.12 to 1.30)	1.14 (1.04 to 1.24)	1.24 (1.14 to 1.35)
All exposures combined	1.22 (1.14 to 1.30)	1.17 (1.05 to 1.31)	1.23 (1.14 to 1.33)	1.16 (1.06 to 1.27)	1.28 (1.17 to 1.39)
**ABSI**					
Individual exposure	1.06 (1.00 to 1.13)	1.10 (1.00 to 1.22)	1.03 (0.95 to 1.12)	1.08 (0.98 to 1.18)	1.05 (0.97 to 1.15)
+ WBC	1.05 (0.99 to 1.12)	1.08 (0.98 to 1.20)	1.02 (0.94 to 1.10)	1.06 (0.97 to 1.16)	1.04 (0.95 to 1.13)
+ WBC + CRP	1.03 (0.97 to 1.10)	1.06 (0.96 to 1.18)	1.00 (0.93 to 1.09)	1.03 (0.94 to 1.13)	1.03 (0.94 to 1.12)
+ WBC + CRP + PLT parameters	1.02 (0.96 to 1.09)	1.05 (0.95 to 1.17)	1.00 (0.92 to 1.08)	1.02 (0.93 to 1.12)	1.02 (0.94 to 1.12)
+ WBC + CRP + PLT param + BMI	1.03 (0.96 to 1.09)	1.05 (0.95 to 1.17)	1.00 (0.93 to 1.09)	1.03 (0.94 to 1.12)	1.03 (0.94 to 1.12)
All exposures combined	1.05 (0.99 to 1.12)	1.07 (0.96 to 1.18)	1.03 (0.95 to 1.12)	1.05 (0.95 to 1.15)	1.05 (0.97 to 1.15)
**HI**					
Individual exposure	0.91 (0.86 to 0.96)	0.88 (0.80 to 0.97)	0.92 (0.86 to 0.99)	0.92 (0.85 to 1.00)	0.90 (0.83 to 0.97)
+ WBC	0.91 (0.86 to 0.96)	0.88 (0.80 to 0.97)	0.92 (0.86 to 0.99)	0.92 (0.85 to 1.00)	0.89 (0.83 to 0.97)
+ WBC + CRP	0.90 (0.85 to 0.96)	0.88 (0.80 to 0.97)	0.92 (0.86 to 0.99)	0.92 (0.84 to 1.00)	0.89 (0.83 to 0.97)
+ WBC + CRP + PLT parameters	0.91 (0.86 to 0.96)	0.89 (0.81 to 0.97)	0.92 (0.86 to 0.99)	0.92 (0.85 to 1.00)	0.90 (0.83 to 0.97)
+ WBC + CRP + PLT param + BMI	0.89 (0.84 to 0.94)	0.87 (0.79 to 0.96)	0.90 (0.84 to 0.97)	0.91 (0.84 to 0.98)	0.88 (0.81 to 0.95)
All exposures combined	0.88 (0.83 to 0.93)	0.87 (0.79 to 0.95)	0.90 (0.83 to 0.96)	0.90 (0.83 to 0.98)	0.87 (0.80 to 0.94)

0.8 1.0 1.2 1.4 (x-axis for each panel)

Legend: + Individual exposures; Adding NEU; Adding all WBC; Adding CRP; Adding PLT or PDW; Adding all PLT parameters; Adding BMI; All exposures combined

Supplementary Figure S3B Sequential addition of exposures – associations of platelet parameters and allometric obesity indices with kidney cancer risk

Supplementary Figure S3 Sequential addition of exposures − legend

ABSI – a body shape index; **BMI** – body mass index; **CI** – confidence interval; **CRP** – C-reactive protein; **HI** – hip index; **HR** – hazard ratio; **MPV** – mean platelet volume; **PDW** – platelet distribution width; **PLT** – platelet count; **SD** – standard deviation; **WBC** – white blood cell (leucocyte) counts (neutrophil count, monocyte count, lymphocyte count as separate variables); **FU<6 years** – cases diagnosed within the first 6 years of follow-up, censoring follow-up at 6 years; **FU≥6 years** – cases diagnosed after 6 years of follow-up, lagging entry time with 6 years and excluding participants with follow-up<6 years.

Cox proportional hazards models stratified by age at recruitment, smoking status and intensity, and sex (except for sex-specific models), and adjusted for height (sex-specific z-scores), alcohol consumption, physical activity, education, diabetes, use of lipid-lowering drugs, hypertension (with or without treatment), use of antiaggregant/anticoagulants, and dietary intake of fruit and vegetables, red and processed meat, and fibre.

Sequential addition of exposures in the order specified for each primary exposure in the plot:

Individual exposures – including each exposure individually (sex-specific z-scores, value minus mean divided by standard deviation, after log-transformation for biomarkers).

Adding NEU – adding neutrophil count.

Adding all WBC – adding all white blood cell (leucocyte) count variables − neutrophil count, monocyte count, lymphocyte count. For leucocyte counts as primary exposures, combining each with the other two.

Adding CRP – adding CRP.

Adding PLT or PDW – adding PLT to models with neutrophil count, CRP, MPV, or PDW as the primary exposures or adding PDW to the model with PLT as the primary exposure.

Adding all PLT parameters – adding all platelet parameters (platelet count, MPV, PDW). For platelet parameters as primary exposures, each was combined with the other two.

Adding BMI – adding BMI. Note that adding BMI to models including ABSI and HI as the primary exposure accounts for minor residual correlations with BMI due to the derivation of the original allometric power coefficients in an external dataset.

All exposures combined – the main multivariable model including all exposures and shown in Figure 2 for leucocyte counts and CRP, Figure 3 for platelet parameters, and Figure 4 for allometric obesity indices.

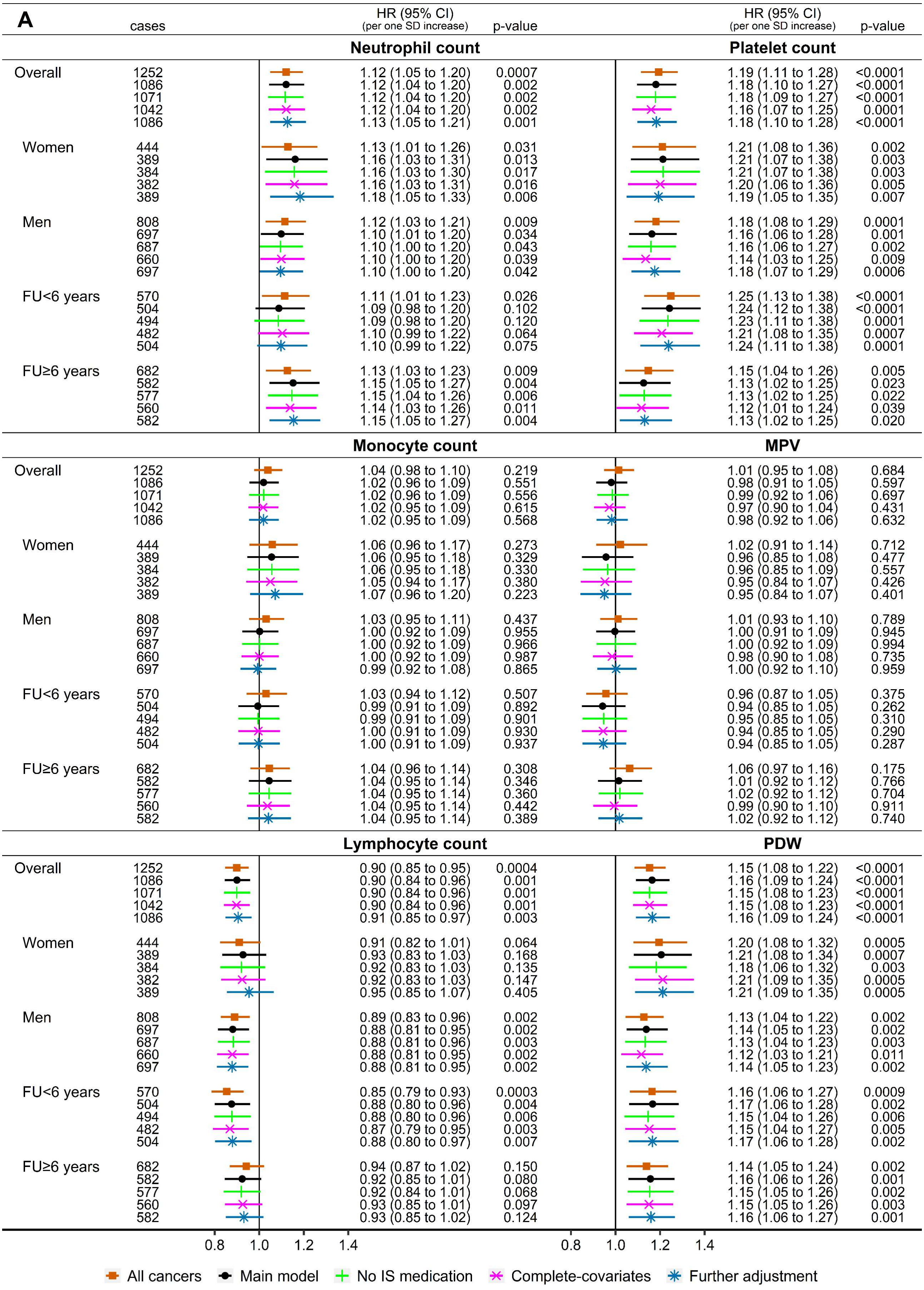

Supplementary Figure S4A Additional sensitivity analyses – associations of leucocyte counts and platelet parameters with kidney cancer risk

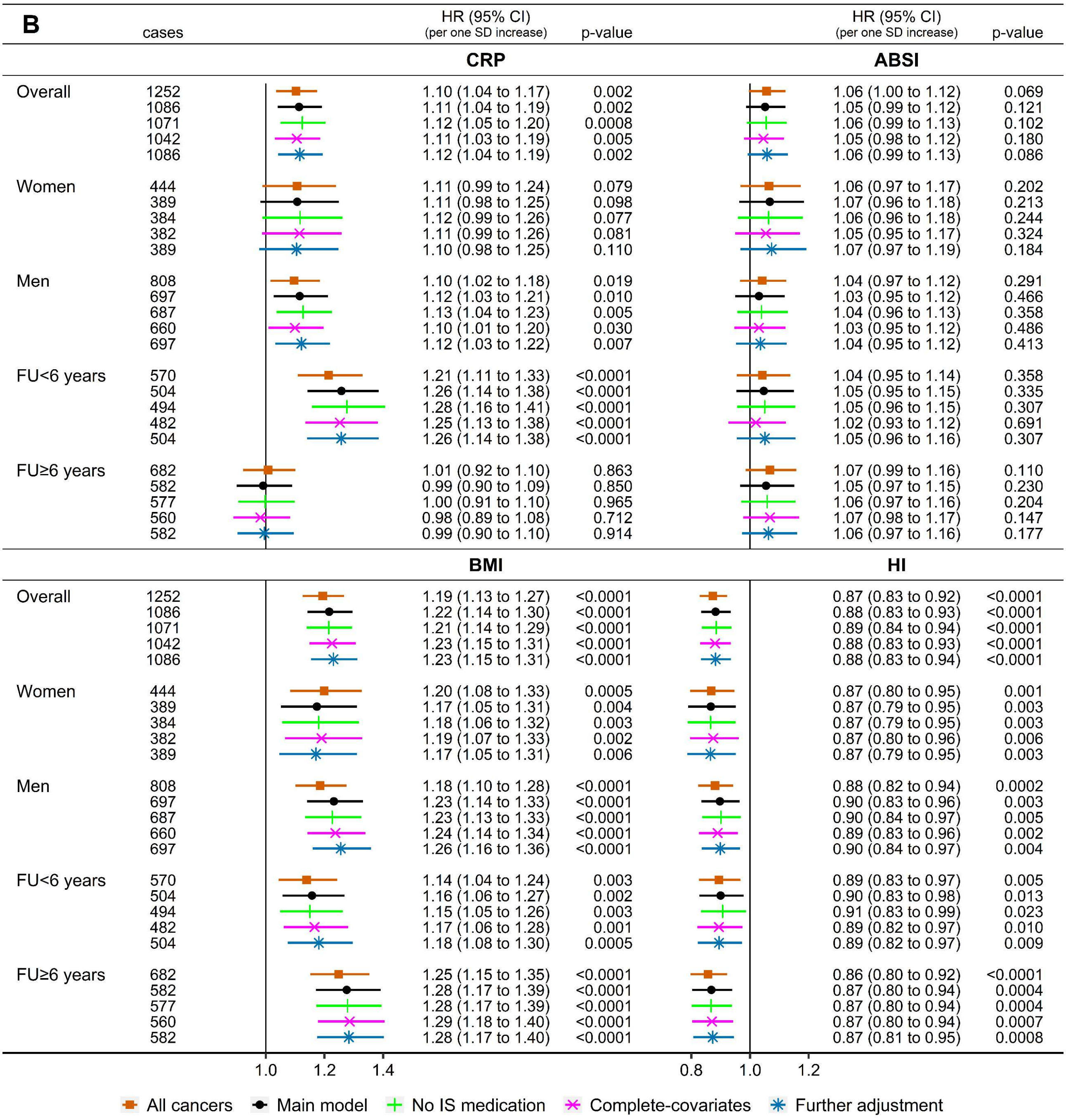

	cases	CRP HR (95% CI) (per one SD increase)	CRP p-value	ABSI HR (95% CI) (per one SD increase)	ABSI p-value
Overall	1252	1.10 (1.04 to 1.17)	0.002	1.06 (1.00 to 1.12)	0.069
	1086	1.11 (1.04 to 1.19)	0.002	1.05 (0.99 to 1.12)	0.121
	1071	1.12 (1.05 to 1.20)	0.0008	1.06 (0.99 to 1.13)	0.102
	1042	1.11 (1.03 to 1.19)	0.005	1.05 (0.98 to 1.12)	0.180
	1086	1.12 (1.04 to 1.19)	0.002	1.06 (0.99 to 1.13)	0.086
Women	444	1.11 (0.99 to 1.24)	0.079	1.06 (0.97 to 1.17)	0.202
	389	1.11 (0.98 to 1.25)	0.098	1.07 (0.96 to 1.18)	0.213
	384	1.12 (0.99 to 1.26)	0.077	1.06 (0.96 to 1.18)	0.244
	382	1.11 (0.99 to 1.26)	0.081	1.05 (0.95 to 1.17)	0.324
	389	1.10 (0.98 to 1.25)	0.110	1.07 (0.97 to 1.19)	0.184
Men	808	1.10 (1.02 to 1.18)	0.019	1.04 (0.97 to 1.12)	0.291
	697	1.12 (1.03 to 1.21)	0.010	1.03 (0.95 to 1.12)	0.466
	687	1.13 (1.04 to 1.23)	0.005	1.04 (0.96 to 1.13)	0.358
	660	1.10 (1.01 to 1.20)	0.030	1.03 (0.95 to 1.12)	0.486
	697	1.12 (1.03 to 1.22)	0.007	1.04 (0.95 to 1.12)	0.413
FU<6 years	570	1.21 (1.11 to 1.33)	<0.0001	1.04 (0.95 to 1.14)	0.358
	504	1.26 (1.14 to 1.38)	<0.0001	1.05 (0.95 to 1.15)	0.335
	494	1.28 (1.16 to 1.41)	<0.0001	1.05 (0.96 to 1.15)	0.307
	482	1.25 (1.13 to 1.38)	<0.0001	1.02 (0.93 to 1.12)	0.691
	504	1.26 (1.14 to 1.38)	<0.0001	1.05 (0.96 to 1.16)	0.307
FU≥6 years	682	1.01 (0.92 to 1.10)	0.863	1.07 (0.99 to 1.16)	0.110
	582	0.99 (0.90 to 1.09)	0.850	1.05 (0.97 to 1.15)	0.230
	577	1.00 (0.91 to 1.10)	0.965	1.06 (0.97 to 1.16)	0.204
	560	0.98 (0.89 to 1.08)	0.712	1.07 (0.98 to 1.17)	0.147
	582	0.99 (0.90 to 1.10)	0.914	1.06 (0.97 to 1.16)	0.177

	cases	BMI HR (95% CI) (per one SD increase)	BMI p-value	HI HR (95% CI) (per one SD increase)	HI p-value
Overall	1252	1.19 (1.13 to 1.27)	<0.0001	0.87 (0.83 to 0.92)	<0.0001
	1086	1.22 (1.14 to 1.30)	<0.0001	0.88 (0.83 to 0.93)	<0.0001
	1071	1.21 (1.14 to 1.29)	<0.0001	0.89 (0.84 to 0.94)	<0.0001
	1042	1.23 (1.15 to 1.31)	<0.0001	0.88 (0.83 to 0.93)	<0.0001
	1086	1.23 (1.15 to 1.31)	<0.0001	0.88 (0.83 to 0.94)	<0.0001
Women	444	1.20 (1.08 to 1.33)	0.0005	0.87 (0.80 to 0.95)	0.001
	389	1.17 (1.05 to 1.31)	0.004	0.87 (0.79 to 0.95)	0.003
	384	1.18 (1.06 to 1.32)	0.003	0.87 (0.79 to 0.95)	0.003
	382	1.19 (1.07 to 1.33)	0.002	0.87 (0.80 to 0.96)	0.006
	389	1.17 (1.05 to 1.31)	0.006	0.87 (0.79 to 0.95)	0.003
Men	808	1.18 (1.10 to 1.28)	<0.0001	0.88 (0.82 to 0.94)	0.0002
	697	1.23 (1.14 to 1.33)	<0.0001	0.90 (0.83 to 0.96)	0.003
	687	1.23 (1.13 to 1.33)	<0.0001	0.90 (0.84 to 0.97)	0.005
	660	1.24 (1.14 to 1.34)	<0.0001	0.89 (0.83 to 0.96)	0.002
	697	1.26 (1.16 to 1.36)	<0.0001	0.90 (0.84 to 0.97)	0.004
FU<6 years	570	1.14 (1.04 to 1.24)	0.003	0.89 (0.83 to 0.97)	0.005
	504	1.16 (1.06 to 1.27)	0.002	0.90 (0.83 to 0.98)	0.013
	494	1.15 (1.05 to 1.26)	0.003	0.91 (0.83 to 0.99)	0.023
	482	1.17 (1.06 to 1.28)	0.001	0.89 (0.82 to 0.97)	0.010
	504	1.18 (1.08 to 1.30)	0.0005	0.89 (0.82 to 0.97)	0.009
FU≥6 years	682	1.25 (1.15 to 1.35)	<0.0001	0.86 (0.80 to 0.92)	<0.0001
	582	1.28 (1.17 to 1.39)	<0.0001	0.87 (0.80 to 0.94)	0.0004
	577	1.28 (1.17 to 1.39)	<0.0001	0.87 (0.80 to 0.94)	0.0004
	560	1.29 (1.18 to 1.40)	<0.0001	0.87 (0.80 to 0.94)	0.0007
	582	1.28 (1.17 to 1.40)	<0.0001	0.87 (0.81 to 0.95)	0.0008

Supplementary Figure S4B Additional sensitivity analyses – associations of CRP and allometric obesity indices with kidney cancer risk

ABSI – a body shape index; **BMI** – body mass index; **CI** – confidence interval; **CRP** – C-reactive protein; **HI** – hip index; **HR** – hazard ratio; **IS** – immunosuppressant; **MPV** – mean platelet volume; **PDW** – platelet distribution width; **SD** – standard deviation; **cases** – number of kidney cancer cases; **p-value** – Wald test for the individual term; **FU<6 years** – cases diagnosed within the first 6 years of follow-up, censoring follow-up at 6 years; **FU≥6 years** – cases diagnosed after 6 years of follow-up, lagging entry time with 6 years and excluding participants with follow-up<6 years.

Supplementary Figure S4 Additional sensitivity analyses – legend (continued)

Cox proportional hazards models including all exposures combined (sex-specific z-scores, value minus mean divided by standard deviation, after log-transformation for biomarkers), stratified by age at recruitment, smoking status and intensity, and sex (except for sex-specific models), and adjusted for height (sex-specific z-scores), alcohol consumption, physical activity, education, diabetes, use of lipid-lowering drugs, hypertension (with or without treatment), use of antiaggregant/ anticoagulants, and dietary intake of fruit and vegetables, red and processed meat, and fibre.

All cancers – considering all primary incident kidney cancers as outcome (irrespective of the order of diagnosis) and continuing follow-up after diagnosis of incident cancer in another location. Exit time for this analysis was defined as the earliest of the date of diagnosis of primary incident kidney cancer, or death, or last complete follow-up (mean follow-up 10.8 years; overall incidence rate 292 per $1*10^6$ person years; 242 per $1*10^6$ person years for follow-up <6 years; 353 per $1*10^6$ person years for follow-up ≥6 years; 388,715 cohort participants with follow-up ≥6 years). Note that all incident kidney cancers with ICD10 code C64 had behavioural code 3 (malignant, primary site) or 5 (malignant, microinvasive), except one with missing behavioural code, which was included in this analysis.

Main model – the main multivariable model (all exposures combined), considering first primary kidney cancer as outcome and censoring at diagnosis first primary cancers in other locations.

No IS medication – as the main model, excluding 3670 cohort participants (0.9% of the main dataset) with self-reported use of immunosuppressants, based on Fields [20003-0/47] "Treatment/ medication code" with the following codes (UK Biobank Coding 4):

1140869848	methotrexate	1140925978	mycophenolate
1140869870	maxtrex 2.5mg tablet	1140925986	cellcept 250mg capsule
1140869930	azathioprine	1141145996	imuran 10mg tablet
1140869932	azamune 50mg tablet	1141173926	sirolimus
1140869940	immunoprin 50mg tablet	1141181020	ciclosporin product
1140869942	berkaprine 50mg tablet	1141200446	mycophenolic acid 180mg gastro-resistant tablet
1140869952	cyclosporin product	1141200450	myfortic 180mg gastro-resistant tablet
1140870016	sandimmun 25mg capsule	2018943436	csa - cyclosporin a
1140884308	hydroxychloroquine	2018943438	cya - cyclosporin a
1140909844	ciclosporin	2038459888	cyclosporin
1140909864	azt - azathioprine	2038459890	cyclosporin a
1140910036	mtx - methotrexate	1140925978	mycophenolate
1140910382	cya - cyclosporin	1140925986	cellcept 250mg capsule
1140911642	tacrolimus	1141145996	imuran 10mg tablet

Total included 392,812 (women 209,565; men 183,247; follow-up ≥6 years 368,904).

Complete-covariates – as the main model, excluding cohort participants with missing any covariate.

Further adjusted – as the main model, further adjusted for all candidate covariates considered individually in **Supplementary Figure S1** as follows: use of non-steroidal anti-inflammatory drugs, paracetamol use, weight gain within the year prior to recruitment, family history of cancer, fasting time, time of blood collection, region of the assessment centre, Townsend deprivation index (tertile cut-offs: −3.22 and −0.84), fish>twice/week, tea>2 cups/day, coffee>4 cups/day, and replacing the stratification by sex with a stratification by a combined variable of sex, menopausal status, and use of hormone replacement therapy (HRT) (categories: men, pre-menopausal women, post/unknown menopause never used HRT, post/unknown menopause past HRT use, post/unknown menopause current HRT use).

References

Numbers correspond to the main document:

18. Christakoudi S, Tsilidis KK, Evangelou E, Riboli E. A Body Shape Index (ABSI), hip index and risk of cancer in the UK Biobank cohort. *Cancer Medicine*. 2021;10(16):5614-28. https://doi.org/10.1002/CAM4.4097

26. Christakoudi S, Tsilidis KK, Gunter MJ, Riboli E. Prospective Associations of Body Composition and Body Shape With the Risk of Developing Pancreatic Cancer in the UK Biobank Cohort. *Cancer Med*. 2025;14(6):e70809. https://doi.org/10.1002/cam4.70809

27. Christakoudi S, Tsilidis KK, Evangelou E, Riboli E. Interactions of platelets with obesity in relation to lung cancer risk in the UK Biobank cohort. *Respir Res*. 2023;24(1):249. https://doi.org/10.1186/s12931-023-02561-9
